# Rab11 activation by Ik2 kinase is required for dendrite pruning in *Drosophila* sensory neurons

**DOI:** 10.1371/journal.pgen.1008626

**Published:** 2020-02-14

**Authors:** Tzu Lin, Hao-Hsiang Kao, Che-Hsuan Chou, Chih-Yu Chou, Yu-Ching Liao, Hsiu-Hsiang Lee

**Affiliations:** Institute of Molecular Medicine, College of Medicine, National Taiwan University, Taipei, Taiwan; Stanford University School of Medicine, UNITED STATES

## Abstract

Neuronal pruning is a commonly observed phenomenon for the developing nervous systems to ensure precise wiring of neural circuits. The function of Ik2 kinase and its downstream mediator, Spindle-F (Spn-F), are essential for dendrite pruning of *Drosophila* sensory neurons during development. However, little is known about how Ik2/Spn-F signaling is transduced in neurons and ultimately results in dendrite pruning. Our genetic analyses and rescue experiments demonstrated that the small GTPase Rab11, especially the active GTP-bound form, is required for dendrite pruning. We also found that *Rab11* shows genetic interactions with *spn-F* and *ik2* on pruning. Live imaging of single neurons and antibody staining reveal normal Ik2 kinase activation in *Rab11* mutant neurons, suggesting that Rab11 could have a functional connection downstream of and/or parallel to the Ik2 kinase signaling. Moreover, we provide biochemical evidence that both the Ik2 kinase activity and the formation of Ik2/Spn-F/Rab11 complexes are central to promote Rab11 activation in cells. Together, our studies reveal that a critical role of Ik2/Spn-F signaling in neuronal pruning is to promote Rab11 activation, which is crucial for dendrite pruning in neurons.

## Author summary

During metamorphosis in *Drosophila*, both the central and peripheral nervous systems undergo substantial neuronal remodeling, such as the cell death of most larval neurons and regeneration of adult neurons, while few larval neurons remain alive and prune their branches. Pruning is a self-destruction program, and thus requires to be tightly controlled within single neurons spatially and temporally during development. Recent studies have shown a strong correlation between pruning and human psychiatric disorders, such as schizophrenia and autism. *Drosophila* sensory neurons that undergo dendrite pruning provide us an opportunity to study the regulatory mechanism of neuronal pruning. Previously, we identified an IKK-related kinase Ik2 that is essential and sufficient for dendrite pruning, and a coiled-coil protein Spindle-F that mediates Ik2-dependent pruning activity in neurons. However, what are the downstream targets of Ik2/Spindle-F signaling in dendrite pruning remains unclear. In this study, we found that the small GTPase Rab11, especially the active GTP-bound form, is required for dendrite pruning in neurons. We further demonstrated that both the Ik2 kinase activity and Ik2/Spindle-F complexes are essential to enhance Rab11 activation in neurons during dendrite pruning.

## Introduction

During the early development of the nervous systems, neurons often extend exuberant branches and make excessive connections in the vicinity of their final targets. To ensure the precise neuronal wiring, further remodeling is required to refine the connections of the nervous systems at later developmental stages. Neuronal pruning, one of the remodeling mechanisms, is a tightly controlled process to eliminate excessive branches and improper connections without causing any cell death. Pruning is a commonly observed event in the developing nervous systems of both vertebrates and invertebrates [1,2]. It has been shown that disrupting developmental pruning in neurons could reduce olfactory plasticity in *C*. *elegans* [3]. Moreover, blocking pruning in *Drosophila* brain mushroom body (MB) γ neurons has been shown to display ectopic connections affecting the olfactory circuits [4], and also abolish short-term courtship memory in adult flies [5]. Thus, any misregulation of pruning activity could severely affect the functions of nervous systems in animals.

Neuronal remodeling occurs in both the central and peripheral nervous systems of *Drosophila* during metamorphosis [6–10]. Most larval neurons die during metamorphosis, but only few survive and undergo neuronal pruning. The peripheral class IV dendritic arborization (C4da) neurons are one type of such neurons that undergo a large-scale dendrite pruning [7–10]. Dendrite pruning of dorsal C4da neurons begins with dendrite severing at the proximal parts of dendrites around 4–6 hours after puparium formation (APF) [11]. Subsequently, the disconnected dendrites become fragmented and removed via phagocytosis by the underlying epidermal cells by 16–18 hours APF [12]. Dendrite pruning is initiated by the steroid hormone ecdysone signaling and its target gene *sox14* in C4da neurons at early pupal stages [7,8,13]. A few other molecules involved in specific cellular activities, such as the ubiquitin-proteasome system [8,14], caspases [15,16], matrix metalloproteases [8], microtubule severing and breakdown proteins [11,17,18], and mediators of dendritic calcium transients [19], were reported to participate in dendrite pruning of C4da neurons. It has also been shown that endocytic pathways play an important role in neuronal pruning in both the central and peripheral nervous systems of *Drosophila* [20,21]. The small GTPase Rab5 is required not only for global endocytosis mediating the degradation of cell adhesion molecule Neuroglian (Nrg) [21], but also for local endocytosis contributing to compartmentalized calcium transients in the dendrite pruning of C4da neurons [22], underlining the crucial roles of Rab GTPases in neuronal pruning. The Rab GTPase proteins alternate between GTP- and GDP-bound states in cells. The binding of GTP or GDP affects the conformations of Rab proteins, and that conformations would determine the interactions of Rab proteins with their downstream effectors. The Rab GTPases family proteins are key players in regulating membrane identity and coordinating vesicular transport between different organelles in cells. The small GTPase Rab11 specifically regulates vesicle trafficking through the recycling endosomes back to the plasma membrane, exocytosis from the trans-Golgi networks and post-Golgi vesicles, and cytokinesis [23].

Previously, we have demonstrated that an IKK-related kinase Ik2 is essential for dendrite pruning of *Drosophila* sensory neurons [11]. We further identified a coiled-coil protein Spindle-F (Spn-F) that acts downstream of Ik2 kinase and mediates Ik2-dependent pruning activity in pupal neurons [24]. To our knowledge, Ik2 kinase is the only known molecule sufficient to induce precocious dendrite severing in larval neurons [11], highlighting the central role of Ik2/Spn-F signaling activity in dendrite pruning. However, the mechanism by which Ik2/Spn-F signaling is transduced and eventually leads to dendrite pruning in C4da neurons remains elusive. To further study the molecular mechanisms of dendrite pruning in C4da neurons, we identified a small GTPase Rab11 as a candidate regulator for dendrite pruning.

In this study, we provide evidence to show that Rab11 plays a critical role in dendrite pruning of *Drosophila* sensory neurons. Our genetic experiments demonstrate that Rab11, the active GTP-bound form in particular, is essential for dendrite pruning in C4da neurons. We further show that *Rab11* displays genetic interactions with *spn-F* and *ik2* on dendrite pruning. Given the normal Ik2 kinase activation in neurons with impaired Rab11 function and the functional connection between Rab11 and Ik2/Spn-F, Rab11 could act downstream of and/or parallel to the Ik2 kinase signaling. Moreover, we found that the formation of Ik2/Spn-F/Rab11 complexes and the kinase activity of Ik2 are both essential to promote Rab11 activation in cells. Taken together, the results from our studies reveal that a critical role of Ik2/Spn-F signaling in neuronal pruning is to promote Rab11 activation, which is crucial for dendrite pruning in C4da neurons.

## Results

### The small GTPase Rab11 is required for dendrite pruning

**Fig 1:**
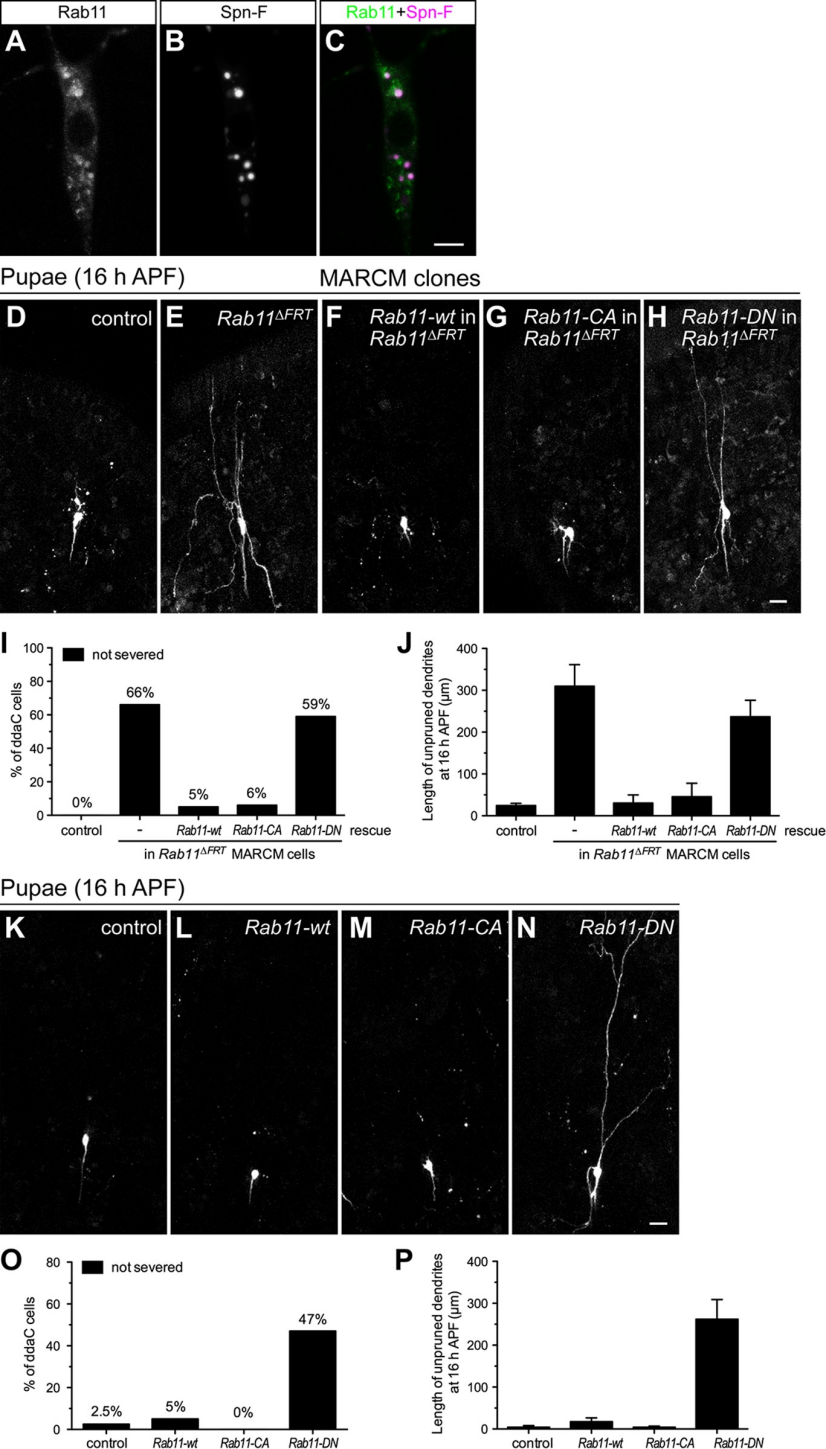
Rab11 and its GTP-bound form are required for dendrite pruning. (A-C) The expression of Rab11-YFP (A) and Spn-F-mCherry (B) were under the control of *ppk-GAL4* in larval ddaC neurons. The merged image is shown in (C). (D-H) Confocal images of MARCM (Mosaic analysis with a repressible cell marker) clones of ddaC neurons at 16 h APF (after puparium formation). The dendrite pruning occurred normally in the control clones (D), but was defective in the *Rab11*^*ΔFRT*^ clones (E). The expression of wt (wild-type) Rab11 (F) or of Rab11-CA (constitutively active) (G), but not Rab11-DN (dominant negative) (H), could rescue the pruning defects in *Rab11*^*ΔFRT*^ clones. (I) Quantification of pruning phenotypes in MARCM clones of ddaC cells at 16 h APF. The percentage of cells was determined by dividing the number of neurons with defective pruning by the total number of cells examined for each genotype; for control clones, n = 21; for *Rab11*^*ΔFRT*^ clones, n = 29; for *Rab11*^*ΔFRT*^ clones rescued with *Rab11-wt*, n = 19; for *Rab11*^*ΔFRT*^ clones rescued with *Rab11-CA*, n = 16; for *Rab11*^*ΔFRT*^ clones rescued with *Rab11-DN*, n = 17. (J) Quantification of the total length of unpruned dendrites in MARCM clones of ddaC cells at 16 h APF. For control clones, n = 21; for *Rab11*^*ΔFRT*^ clones, n = 26; for *Rab11*^*ΔFRT*^ clones rescued with *Rab11-wt*, n = 19; for *Rab11*^*ΔFRT*^ clones rescued with *Rab11-CA*, n = 16; for *Rab11*^*ΔFRT*^ clones rescued with *Rab11-DN*, n = 11. (K-N) The ddaC neurons were marked with *ppk-GAL4* and *UAS-mCD8GFP*. At 16 h APF, the dendrites of ddaC neurons were pruned normally in the control cells (K), the ones with *Rab11-wt* overexpression (L), and the ones with *Rab11-CA* over-expression (M), but not in the ones with *Rab11-DN* overexpression (N). (O) Quantification of pruning phenotypes in neurons expressing *Rab11-wt*, *Rab11-CA* and *Rab11-DN*. For wild-type control, n = 80; for *Rab11-wt* overexpression, n = 100; for *Rab11-CA* over-expression, n = 90; for *Rab11-DN* overexpression, n = 119. (P) Quantification of the total length of unpruned dendrites in neurons expressing *Rab11-wt*, *Rab11-CA* and *Rab11-DN*. For wild-type control, n = 40; for *Rab11-wt* overexpression, n = 40; for *Rab11-CA* over-expression, n = 20; for *Rab11-DN* overexpression, n = 49. Data are mean±SEM in (J, P). Scale bars, 5 μm in (C); 20 μm in (H, N).

Our previous observation that Spn-F proteins exhibited a punctate pattern in the cytosol of larval C4da neurons [24] (Fig 1B) raised the possibility that these punctate Spn-F proteins might associate with unidentified cellular compartments in cells. To examine this possibility, we co-expressed various GFP/YFP-labeled organelle markers, such as Cnn-GFP, Mito-GFP, Golgi-GFP, Grasp65-GFP, KDEL-GFP and Rab(s)-YFP with Spn-F-mCherry in larval C4da neurons, and examined which markers might co-localize with Spn-F-mCherry (Fig 1A–1C and S1 Fig). After investigation, we found some Rab11-YFP signals co-localized with Spn-F-mCherry puncta in larval neurons (Fig 1A–1C), suggesting that Spn-F might coincide with Rab11 in certain cellular sites. Since *spn-F* is required for dendrite pruning of C4da neurons [24], we then asked whether *Rab11* also plays a role in dendrite pruning of *Drosophila* sensory neurons. To test this possibility, we expressed *Rab11* double-stranded RNAs (dsRNAs) under the control of C4da-specific *ppk-GAL4* to knockdown the endogenous Rab11 protein expression in cells by RNA interference (RNAi). We observed pruning defects in *Rab11* RNAi neurons, whose primary dendrites were found connected to the soma, at 16 h APF (S2B–S2D Fig) [25], while the wild-type neurons had pruned their dendrites (S2A, S2C and S2D Fig). To rule out the non-specific effects of *UAS-Rab11-dsRNAs* line in dendrite pruning, we examined the pruning phenotypes in C4da neurons of *UAS-Rab11-dsRNAs* line alone, without the driver *ppk-GAL4*, and did not observe pruning defects (S2C and S2D Fig). It suggests that the impaired dendrite pruning is indeed caused by *Rab11-dsRNAs* expression driven by *ppk-GAL4* in C4da neurons. Moreover, we assayed the knockdown efficiency of *UAS-Rab11-dsRNAs* line by expressing it with Rab11-GFP as a reporter in larval C4da neurons and measuring the fluorescent intensity of GFP proteins in the cell bodies. While comparing to the control cells expressing *Luciferase-dsRNAs* (S2E Fig), we found that the knockdown efficiency of Rab11-GFP proteins could reach to 85% in the soma of neurons expressing *Rab11-dsRNA*s (S2F and S2G Fig).

To further verify the requirement of *Rab11* in C4da neurons during dendrite pruning, a null allele of *Rab11*, *Rab11*^*ΔFRT*^ [26], was used in mosaic analysis with a repressive cell marker (MARCM) technique to generate homozygous *Rab11* mutant clones in otherwise heterozygous background by mitotic recombination [27]. The primary dendrites of C4da neurons were found still attached to the cell bodies of *Rab11*^*ΔFRT*^ MARCM clones (Fig 1E) [25], but not to the ones of control clones (Fig 1D), at 16 h APF. Furthermore, these pruning defects could be successfully rescued by expressing the wild-type (wt) Rab11 in the C4da neurons of *Rab11*^*ΔFRT*^ MARCM clones (Fig 1F, 1I and 1J), indicating that the impaired pruning is indeed caused by a loss-of-function mutation in *Rab11*. Like other small GTPases, Rab11 alternates between active GTP- and inactive GDP-bound states, which enables Rab11 to interact with different effectors in cells. To determine which form of Rab11 is required for dendrite pruning of C4da neurons in pupae, we generated transgenic fly lines expressing either the constitutively active (CA) Rab11 (Q70L) proteins that have defective GTPase activity, or the dominant negative (DN) Rab11 (S25N) proteins that have defects in GTP binding [28]. Overexpression of Rab11-wt or -CA proteins in C4da neurons did not result in pruning defects (Fig 1L, 1M, 1O and 1P), which was similar to the control cells (Fig 1K, 1O and 1P); however, overexpression of Rab11-DN did cause defective pruning (Fig 1N–1P). Moreover, the expression of Rab11-CA, not Rab11-DN, could successfully rescue dendrite pruning defects in the C4da neurons of *Rab11*^*ΔFRT*^ MARCM clones (Fig 1G–1J), indicating that the active GTP-bound Rab11 proteins are essential for dendrite pruning. Taken together, these results demonstrate that Rab11 and its active GTP-bound form are required for dendrite pruning of *Drosophila* C4da neurons.

### Rab11 GTPases play roles in both the dendrite morphogenesis of larval neurons and the dendrite pruning of pupal neurons

**Fig 2:**
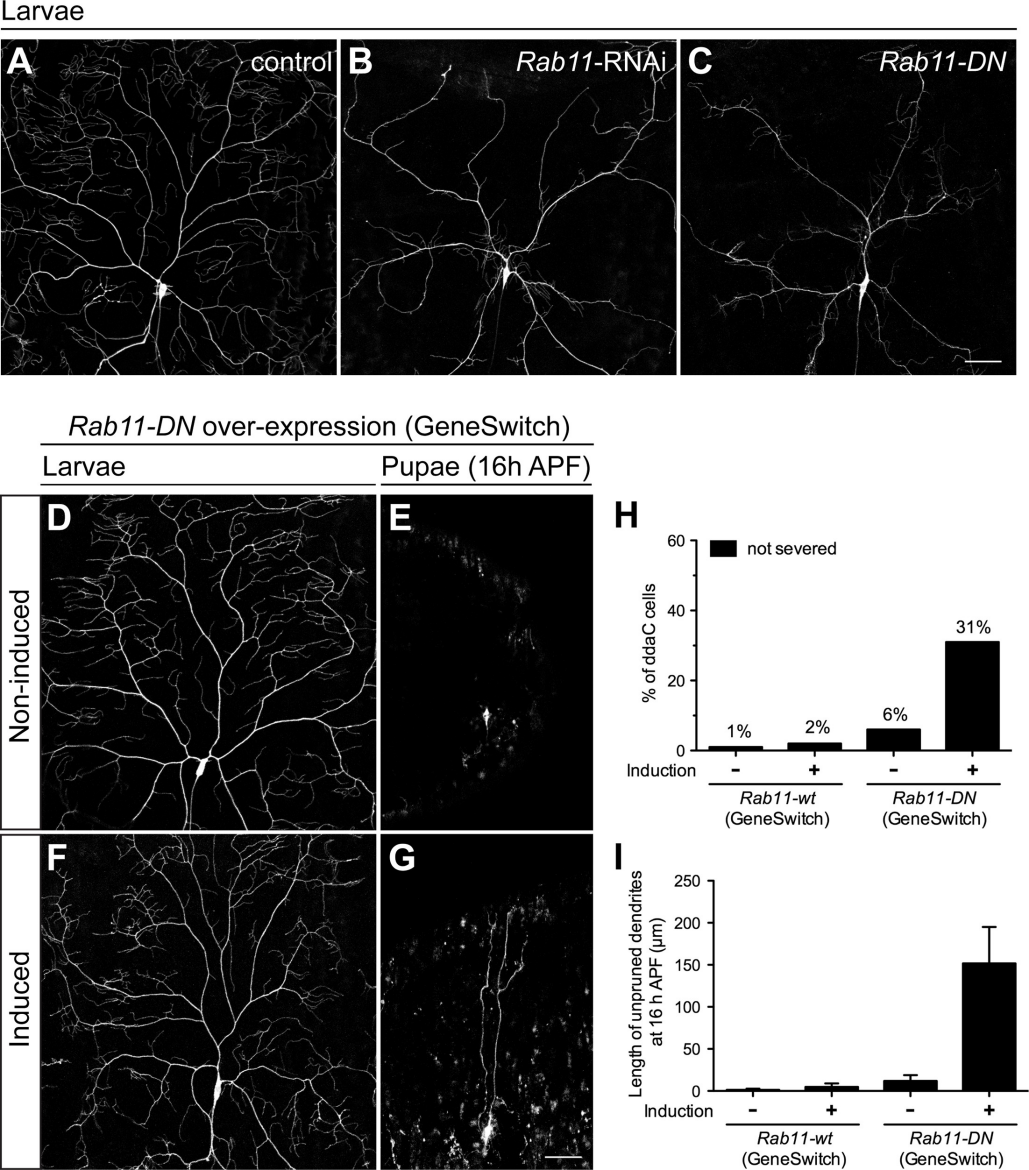
Rab11 plays roles in the larval dendrite morphogenesis and the pupal dendrite pruning. (A-G) The larval ddaC neurons were visualized with *ppk-CD4-tdTomato* expression. The abnormal dendrite morphology with reduced branches was observed in ddaC cells with *Rab11-dsRNAs* (B) and *Rab11-DN* (C) expression under the control of *ppk-GAL4*, compared to the control cells with *Luciferase-dsRNAs* expression (A). (D-G) The *Rab11-DN* expression is driven by *GeneSwitch-GAL4* (*GSG2295-GAL4*), which can be activated by mifepristone. The dendritic morphology was normal in ddaC neurons of late third instar larvae without induction (D) and with induction (F) at 96 h AEL. At 16 h APF, the dendrites were pruned normally in non-induced neurons (E), but remained attached to the induced neurons (G). (H) Quantification of pruning phenotypes in neurons for GeneSwitch experiments at 16 h APF. The percentage of cells was determined by dividing the number of neurons with defective pruning by the total number of cells examined for each genotype; for GeneSwitch expression of *Rab11-wt*, non-induction: n = 80, induction: n = 90; for GeneSwitch expression of *Rab11-DN*, non-induction: n = 110, induction: n = 110. (I) Quantification of the total length of unpruned dendrites in neurons for GeneSwitch experiments at 16 h APF. For GeneSwitch expression of *Rab11-wt*, non-induction: n = 80, induction: n = 50; for GeneSwitch expression of *Rab11-DN*, non-induction: n = 17, induction: n = 32. RNAi, RNA interference. Data are mean±SEM. Scale bars, 50 μm.

While performing *Rab11* RNAi in neurons, we noticed that *Rab11* RNAi also caused abnormal dendritic morphology with reduced dendritic branches in larval C4da neurons (Fig 2B) [25], compared to the control ones with *Luciferase* RNAi (Fig 2A). The similar phenotypes with decreasing dendritic branches were observed not only in the larval neurons with *Rab11-DN* expression (Fig 2C), but also in the ones of *Rab11*^*ΔFRT*^ MARCM clones (S3B Fig). Notably, these abnormal dendrite phenotypes observed in the larval neurons of *Rab11*^*ΔFRT*^ MARCM clones could be completely rescued by expressing either Rab11-wt (S3C Fig) or Rab11-CA (S3D Fig), but not Rab11-DN proteins (S3E Fig). These findings indicate that Rab11 GTPase is crucial for the dendrite morphogenesis in C4da neuron at larval stages.

Since Rab11 has a role in dendrite development, it is possible that the pruning defects we observed in pupal *Rab11* mutant neurons are indirectly due to the abnormal dendrite morphogenesis in larval neurons, not due to the lack of Rab11 function in pupal neurons during pruning. To examine this possibility, we applied the GeneSwitch GAL4 system [29] in C4da neurons to disrupt Rab11 function by inducing Rab11-DN expression only at late larval stages, without affecting dendrite morphogenesis at early stages. In the GeneSwitch system, the GAL4-progesterone-receptor fusion proteins could be activated by binding to mifepristone, which can be administered by feeding. To avoid abnormal dendrite development by interrupting Rab11 function at early larval stages, we transferred the late third instar larvae from regular food to mifepristone-containing food at 96 hours after egg laying (AEL), and fed them for about 24 hours prior to pupation. After nearly 24-hour feeding with mifepristone to induce Rab11-DN expression, we did not observe abnormal dendritic morphology of C4da neurons in the induced larvae (Fig 2F), which is comparable to the ones in non-induced control group (Fig 2D). However, we did observe dendrite pruning defects in C4da neurons with Rab11-DN expression of induced larvae at 16 h APF (Fig 2G–2I), as compared to the control neurons of non-induced animals (Fig 2E, 2H and 2I). It reveals that Rab11 GTPase is indeed required for the dendrite pruning in C4da neurons at pupal stages. To examine whether mifepristone might have non-specific effects in pruning, we used GeneSwitch system to express Rab11-wt proteins in C4da neurons as a control, since the C4da neurons with Rab11-wt overexpression pruned their dendrites normally (Fig 1L, 1O and 1P). After inducing Rab11-wt expression by mifepristone, we observed normal dendrite pruning in C4da neurons at 16 h APF (Fig 2H and 2I), suggesting that feeding larvae with mifepristone does not affect dendrite pruning of C4da neurons in pupae, and further confirming that the function of Rab11 is required for dendrite pruning. Together, our findings demonstrate that Rab11 GTPase not only has a role in the dendrite morphogenesis of C4da neurons at larval stages, but also plays a crucial role in the dendrite pruning of neurons at pupal stages.

### *Rab11* shows genetic interactions with *spn-F* and *ik2* on dendrite pruning

**Fig 3:**
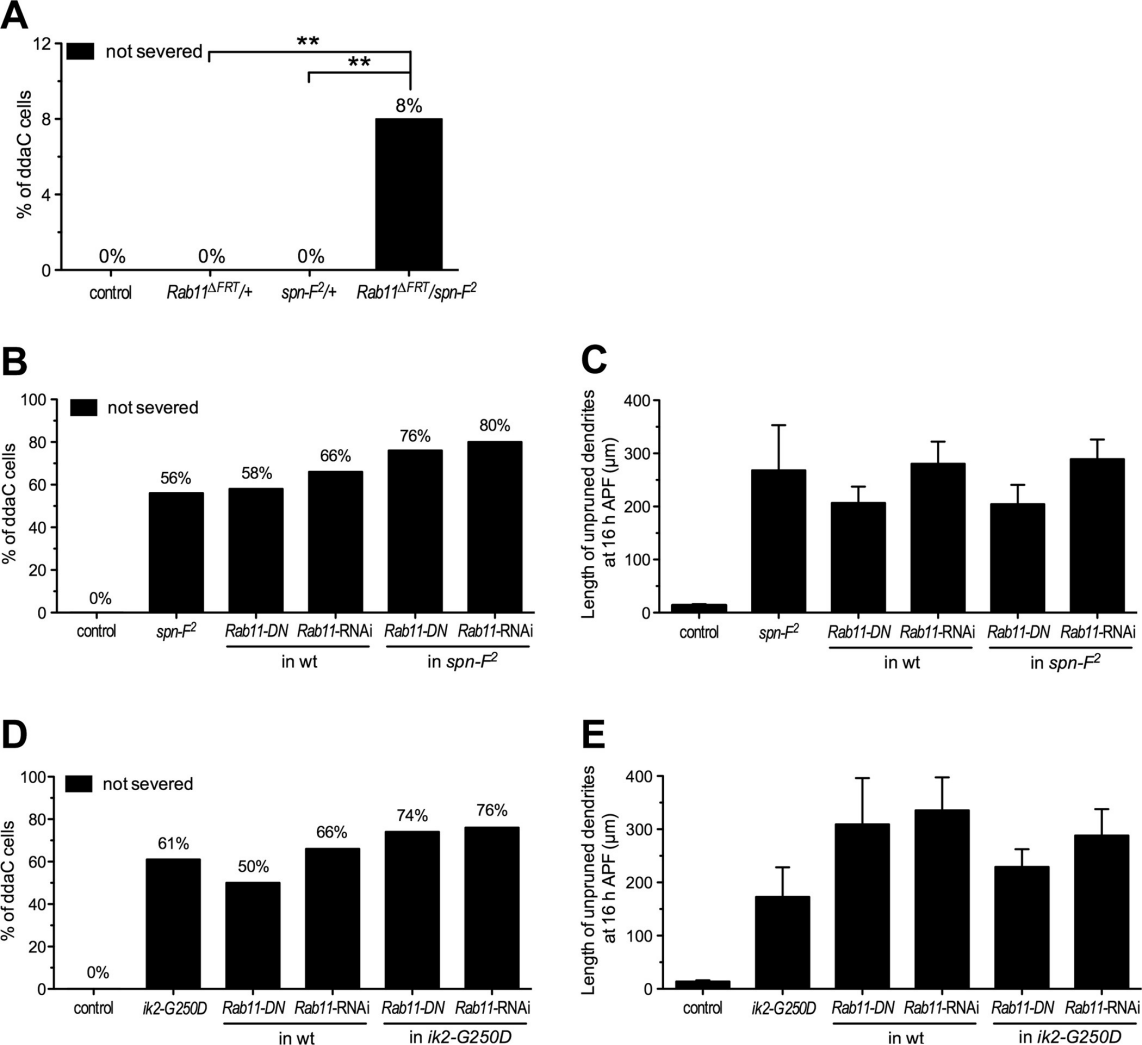
*Rab11* shows genetic interactions with *spn-F* and *ik2* on dendrite pruning. (A) Quantification of dendrite pruning phenotypes in ddaC neurons at 16 h APF showed a genetic interaction between *Rab11* and *spn-F*. The percentage of cells was determined by dividing the number of neurons with defective pruning by the total number of cells examined for each genotype; for the control, n = 90; for *Rab11*^*ΔFRT*^/+, n = 100; for *spn-F*^*2*^/+, n = 100; for *Rab11*^*ΔFRT*^/*spn-F*^*2*^, n = 120. Statistical analysis was performed with Fisher’s exact test. **, p = 0.0055. (B) Quantification of pruning phenotypes in ddaC neurons at 16 h APF. For the control, n = 60; for *spn-F*^*2*^ mutants, n = 100; for *Rab11-DN* overexpression in wild type (wt), n = 50; for *Rab11* RNAi in wt, n = 110; for *Rab11-DN* overexpression in *spn-F*^*2*^ mutants, n = 99; for *Rab11* RNAi in *spn-F*^*2*^ mutants, n = 100. (C) Quantification of the total length of unpruned dendrites in ddaC neurons at 16 h APF. For the control, n = 20; for *spn-F*^*2*^ mutants, n = 21; for *Rab11-DN* overexpression in wild type (wt), n = 45; for *Rab11*-RNAi in wt, n = 23; for *Rab11-DN* overexpression in *spn-F*^*2*^ mutants, n = 17; for *Rab11*-RNAi in *spn-F*^*2*^ mutants, n = 26. (D) Quantification of pruning phenotypes in ddaC neurons at 16 h APF. For the control, n = 70; for *ik2-G250D* overexpression in wt, n = 90; for *Rab11-DN* overexpression in wt, n = 100; for *Rab11*-RNAi in wt, n = 90; for *Rab11-DN* and *ik2-G250D* overexpression, n = 80; for *Rab11*-RNAi with *ik2-G250D* overexpression, n = 100. (E) Quantification of the total length of unpruned dendrites in ddaC neurons at 16 h APF. For the control, n = 20; for *ik2-G250D* overexpression in wt, n = 10; for *Rab11-DN* overexpression in wt, n = 20; for *Rab11*-RNAi in wt, n = 18; for *Rab11-DN* and *ik2-G250D* overexpression, n = 30; for *Rab11*-RNAi with *ik2-G250D* overexpression, n = 25.

Given that both *Rab11* and *spn-F* mutant neurons showed similar pruning phenotypes, we sought to explore a potential genetic interaction between these two genes on dendrite pruning of C4da neurons. To test this possibility, we performed genetic studies by analyzing the pruning phenotypes of C4da neurons in the heterozygotes of either *Rab11* null mutant (*Rab11*^*ΔFRT*^/+) or *spn-F* null mutant (*spn-F*^*2*^/+) [30], as well as in the mutants with *Rab11* and *spn-F* heterozygous combination (*Rab11*^*ΔFRT*^/*spn-F*^*2*^). We did not observe pruning defects in the pupal C4da cells of either *Rab11* or *spn-F* heterozygous mutants (Fig 3A). However, we detected a few number of C4da neurons showing pruning defects in the mutants with heterozygous combination of *Rab11* and *spn-F* (Fig 3A). To confirm this result, we repeated this experiment three times, and consistently observed a few but significant number of C4da neurons exhibiting pruning defects at 16 h APF (the percentage of non-severed neurons for three experiments: 8%, n = 100; 12%, n = 100; 8.2%, n = 110). These results suggest a genetic interaction between *Rab11* and *spn-F* genes on dendrite pruning of C4da neurons. To further investigate the genetic interaction between *Rab11* and *spn-F*, we analyzed the pruning phenotypes of C4da neurons in *Rab11* and *spn-F* single and double mutants. Comparing to the pruning phenotypes in single mutant neurons of either *spn-F* homozygotes or *Rab11* mutants with *Rab11-DN* or -*dsRNAs* expression, we found more C4da neurons in *spn-F* and *Rab11* double mutants showing dendrite pruning defects (Fig 3B and 3C). Since both Ik2 kinase and Spn-F are in the same pathway for dendrite pruning of C4da neurons [24], we also examined the pruning phenotypes in *Rab11* and *ik2* single and double mutants. We observed more C4da neurons exhibiting pruning defects in *ik2* and *Rab11* double mutants than in single *ik2* mutants with kinase-dead *ik2-G250D* expression [31] and single *Rab11* mutants with *Rab11-DN* or -*dsRNAs* expression (Fig 3D and 3E). It confirmed a genetic interaction between *Rab11* and *ik2/spn-F*, and a functional connection between Rab11 proteins and Ik2/Spn-F signaling on dendrite pruning of C4da neurons.

**Fig 4:**
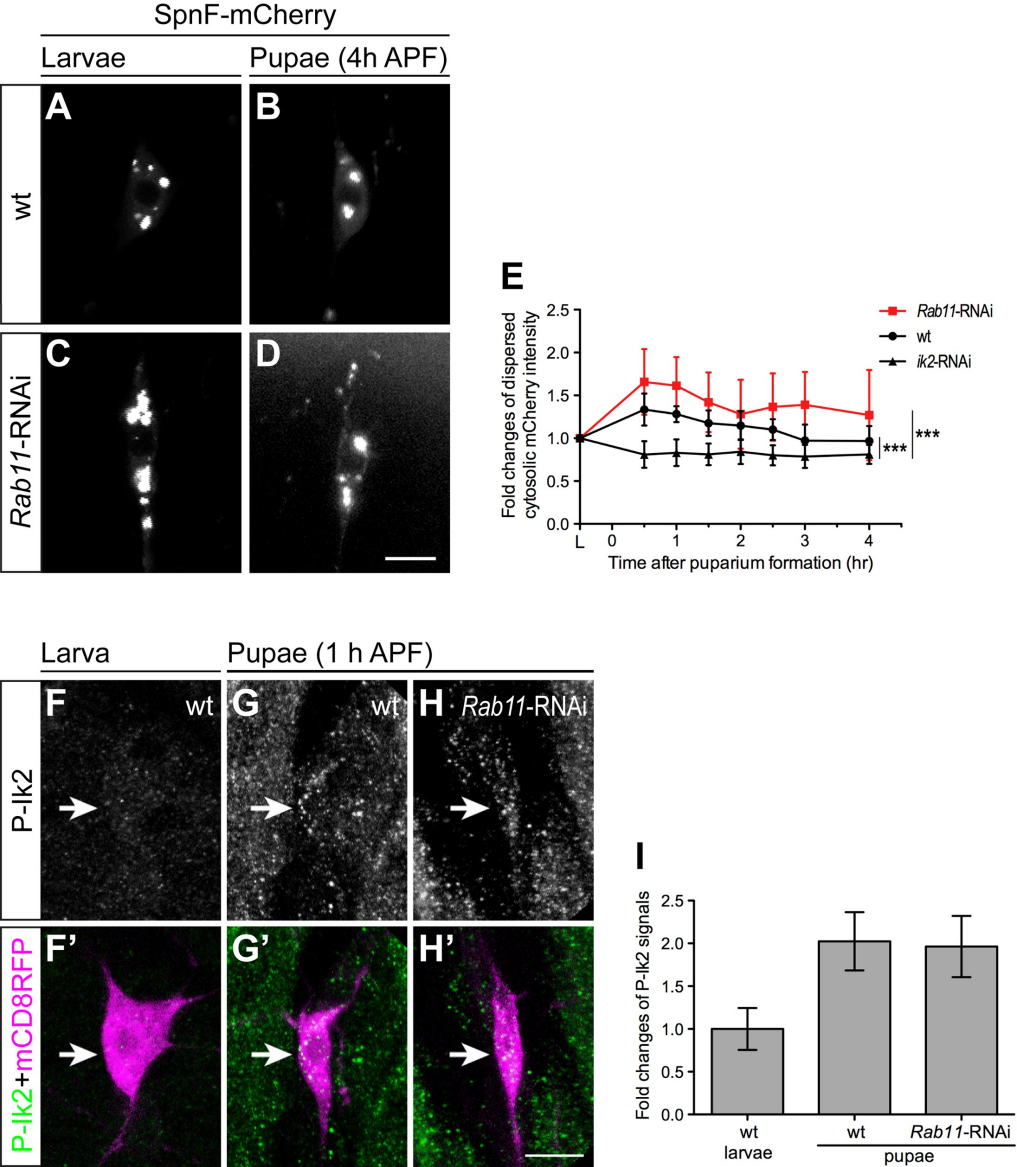
Normal Ik2 activation in *Rab11* mutant pupal neurons. (A-D) The expression of Spn-F-mCherry driven by *ppk-GAL4* in the same wild-type (wt) ddaC cells was shown in larvae (A) and in pupae (B), and in the same *Rab11*-RNAi (RNA interference) neurons was shown in larvae (C) and in pupae (D). The Spn-F-mCherry displayed punctate patterns in the cytosol of both wild-type (A) and *Rab11*-RNAi (C) neurons in larvae. At 4 h APF (after puparium formation), the number and the size of Spn-F-mCherry puncta became decreased in neurons of wild-type (B) and *Rab11*-RNAi (D) pupae. (E) The average fluorescent intensity of dispersed cytosolic mCherry signals was measured in the same neurons of larvae and at various time points of pupae in wild-type, *Rab11*-RNAi and *ik2*-RNAi mutants. The fold changes were determined by dividing the average cytosolic fluorescent intensity of mCherry signals in pupal neurons by that in the same larval cells, which was assigned as 1. For wt, n = 9; for *Rab11*-RNAi, n = 9; for *ik2*-RNAi, n = 9. Data were analyzed by two-way ANOVA (***, p<0.001.). (F’-H’) The ddaC neurons were identified by *ppk-GAL4* and *UAS-mCD8RFP*. (F-H) The activated Ik2 signals were detected by antibodies against phosphorylated Ik2 (P-Ik2). The strong P-Ik2 signals were observed in wild-type and *Rab11*-RNAi pupal C4da neurons at 1 h APF (G, H), but not in wild-type larval neurons (F). (I) The average P-Ik2 signal intensity was measured in the cell body of ddaC neurons in (F-H). For wild-type larval neurons, n = 15; for wild-type pupal neurons, n = 8; for *Rab11*-RNAi pupal neurons, n = 9. The fold changes were determined by dividing the cytosolic fluorescent intensity of P-Ik2 signals in neurons by the average P-Ik2 signal in larval cells, which is assigned as 1. Error bars show SD. Scale bar, 10 μm in (D, H’).

To gain more mechanistic insight into the genetic interaction between *Rab11* and *spn-F* genes, we next asked whether *Rab11* mutation could affect Ik2 kinase activation, as Ik2 kinase is essential for dendrite pruning in C4da neurons and acts upstream of Spn-F [24]. Since Spn-F punctum redistribution in early pupal neurons depends on Ik2 kinase activity [24], the dispersion of Spn-F puncta could be an indicator of Ik2 kinase activation. To detect Ik2 kinase activation in C4da neurons, we performed live-cell imaging to monitor Spn-F-mCherry distribution in the same cells of the wild type and *Rab11* mutants from larvae to early pupae. Spn-F-mCherry formed puncta normally in the larval C4da neurons of both the wild type and *Rab11* mutants (Fig 4A and 4C). While tracking Spn-F-mCherry puncta in the same neurons from larvae (Fig 4A and 4C) to pupae (Fig 4B and 4D), we noticed that the number and sizes of Spn-F puncta decreased in the pupal cells of both the wild type (Fig 4B) and *Rab11* mutants (Fig 4D) at 4 h APF, suggesting that *Rab11* mutation does not affect the Ik2 kinase activation in C4da neurons. To further examine Ik2 kinase activity in *Rab11* mutant neurons, we measured the averaged fluorescent intensity of dispersed cytosolic Spn-F-mCherry signals in the soma, and quantified the fold changes of these signals in the same cells from larvae to early pupae at various time points. Consistent with our previous study using Spn-F-GFP [24], we observed an increase of dispersed cytosolic mCherry signals shortly after pupation in wild-type C4da neurons, but not in *ik2* RNAi cells (Fig 4E). It validated that the prompt increase of dispersed Spn-F-mCherry signals in early pupal neurons depends on Ik2 kinase. Similar to the wild-type C4da neurons, we detected a timely increase of dispersed mCherry signals in the cytosol of *Rab11* RNAi cells soon after pupation (Fig 4E), indicating that Ik2 kinase could be activated normally in *Rab11* mutant neurons. Finally, to detect the activated Ik2 kinase in C4da neurons, we performed staining with antibodies against Ik2 phosphorylation on serine 175 (P-Ik2), which is essential for its activation [32]. Consistent with our previous study [24], the P-Ik2 signals are weak in wild-type larval neurons (Fig 4F and 4I), but strong in pupal neurons (Fig 4G and 4I). Moreover, in the pupal C4da neurons with *Rab11-dsRNAs* expression, we could detect P-Ik2 signals (Fig 4H and 4I), which are comparable to the ones in wild-type pupal neurons (Fig 4G and 4I), confirming the normal Ik2 kinase activation in *Rab11* mutant neurons. Taken together, given the normal Ik2 kinase activation in *Rab11* mutant neurons, the functional connection between Rab11 and Ik2/Spn-F could be downstream of and/or parallel to Ik2 kinase/Spn-F signaling in C4da neurons during dendrite pruning.

### Rab11 forms complexes with Ik2/Spn-F proteins in cells

**Fig 5:**
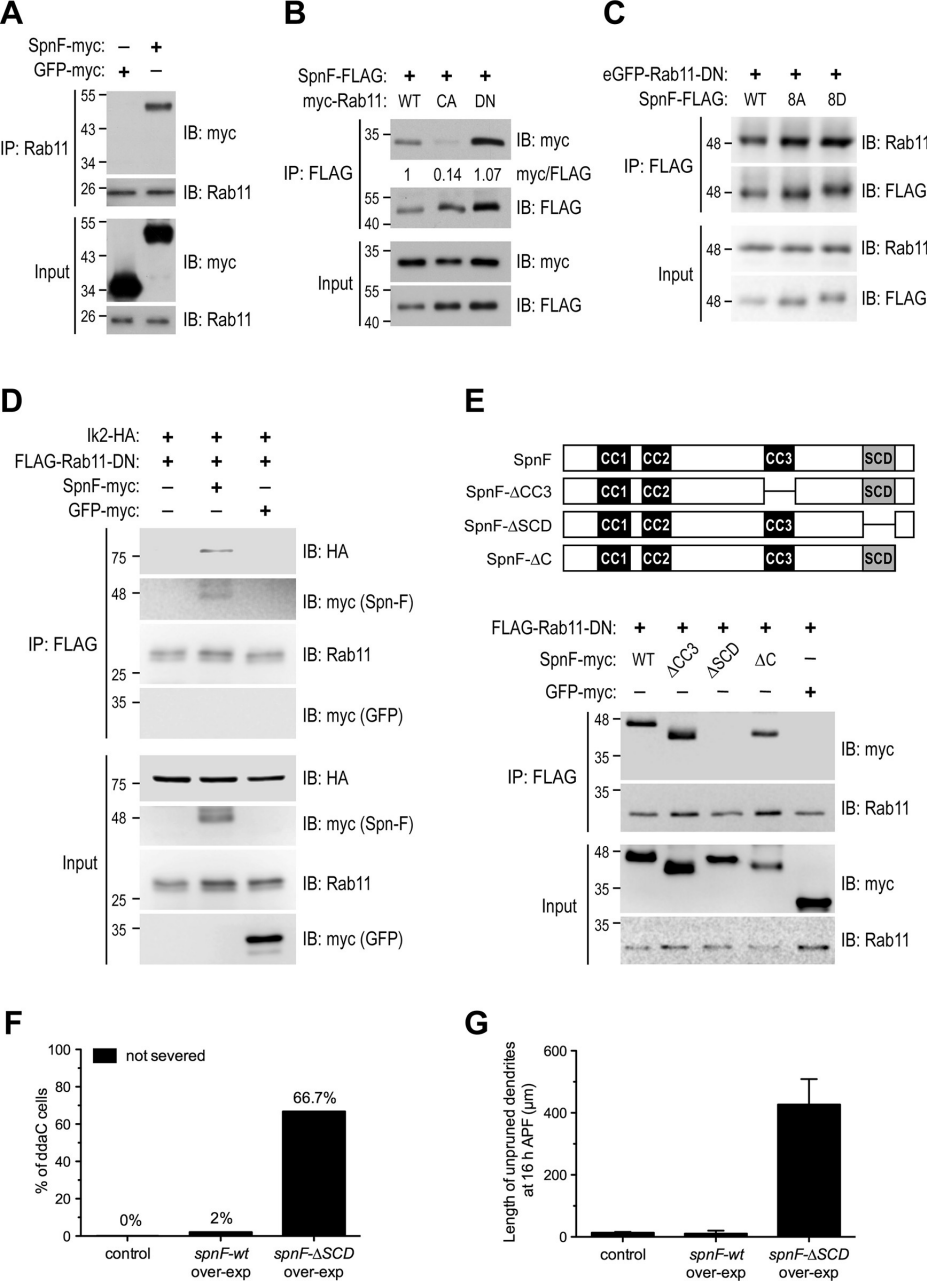
The interaction of Rab11 with Spn-F and Ik2 kinase in cells. (A) The co-IP (co-immunoprecipitation) experiments were performed with lysates from S2 cells expressing Spn-F or GFP as a control to show the specific interaction between Spn-F and endogenous Rab11 proteins. (B) The co-IP experiments with lysates from cells expressing either Rab11-wt, -CA or -DN revealed a preferred interaction between Spn-F and Rab11-DN. The myc/FLAG ratio (by ImageJ) indicates the relative amounts of myc-Rab11 protein associated with Spn-F-FLAG protein in co-IP experiments with anti-FLAG antibody. (C) The co-IP experiments showed no preference of DN-Rab11 for wt-, 8A (phospho-deficient)- and 8D (phospho-mimetic)-Spn-F. (D) The co-IP experiments showed that the formation of Ik2/Spn-F/Rab11 ternary complexes depends on the presence of Spn-F. (E) The co-IP experiments showed that the SCD domain of Spn-F is required for Spn-F/Rab11 interaction. (F) Quantification of pruning defects in neurons at 16 h APF. The percentage of ddaC neurons showing pruning defects among the total number of neurons examined. For wild-type control, n = 60; for *spnF-wt* overexpression, n = 38; for *spnF-ΔSCD* overexpression, n = 90. (G) Quantification of the total length of unpruned dendrites in neurons at 16 h APF. For wild-type control, n = 20; for *spnF-wt* overexpression, n = 38; for *spnF-ΔSCD* overexpression, n = 21. Data are mean±SEM. IB, immunoblotting. over-exp, overexpression.

Considering the co-localization of Rab11-YFP signals and Spn-F-mCherry puncta in larval neurons (Fig 1A–1C), we hypothesized that Rab11 proteins might have a physical interaction with Spn-F proteins in cells. To test this hypothesis, we first performed co-immunoprecipitation (co-IP) with anti-Rab11 antibodies in *Drosophila* S2 cells expressing Spn-F-myc or control GFP-myc. In S2 cell lysates, we could detect the signals of Spn-F-myc, but not the control GFP-myc, in complexes with endogenous Rab11 proteins (Fig 5A), suggesting that Spn-F could associate with endogenous Rab11 proteins in cells. Additionally, we noticed that only some of the Rab11-YFP signals showed co-localization with Spn-F-mCherry (Fig 1A–1C), implying that Spn-F might prefer to interact with certain forms of Rab11 proteins. The small GTPase Rab11 exists in two forms, the active GTP-bound and the inactive GDP-bound forms. To identify which form of Rab11 proteins might have a stronger interaction with Spn-F, we utilized Rab11-CA and Rab11-DN to imitate the active and inactive forms of Rab11, respectively, in co-IP experiments. We found that Spn-F proteins have a stronger interaction with Rab11-DN than with Rab11-wt and -CA proteins in S2 cells (Fig 5B), indicating that Spn-F proteins prefer to associate with the inactive GDP-bound form of Rab11. To further verify whether this is also true in neurons, we examined the co-localization of Spn-F-mCherry with either eGFP-labeled Rab11-DN or -CA in larval C4da cells. Although the signals of eGFP-Rab11-DN tend to be more diffusive than that of eGFP-Rab11-CA in larval C4da neurons, we still observed more Spn-F puncta of small size (its diameter < 0.6 μm) colocalized with Rab11-DN (S4A, S4B and S4E Fig) than with Rab11-CA (S4C–S4E Fig). It confirmed that Spn-F proteins prefer to associate with the inactive GDP-bound Rab11 in C4da neurons. In that case, we decided to use Rab11-DN, instead of Rab11-wt, to study the interaction between Rab11 and Spn-F in the experiments afterwards.

It is known that Spn-F becomes phosphorylated by Ik2 kinase in C4da neurons during dendrite pruning [24]. To examine whether Spn-F phosphorylation by Ik2 kinase may affect the interaction between Spn-F and Rab11, we employed two Spn-F mutants, Spn-F-8A and Spn-F-8D [24]. Spn-F-8A, a phospho-deficient mutant, whose eight serines were replaced by alanines, is used to disrupt Ik2 kinase phosphorylation on Spn-F. Spn-F-8D, a phospho-mimetic mutant with the substitution of aspartic acids for serines, is used to simulate the effects of Ik2 kinase phosphorylation on Spn-F. Our results showed that Rab11-DN displays similar interactions with both Spn-F-8A and Spn-F-8D, as with the wild-type Spn-F in cells (Fig 5C), suggesting that the phosphorylation of Spn-F by Ik2 kinase does not affect the interaction between Spn-F and Rab11 proteins.

Since Spn-F could interact with Ik2 [24] and Rab11 (Fig 5A), we next asked whether Ik2, Spn-F and Rab11 might form complexes in cells. To test this possibility, we performed co-IP experiments in S2 cells. Interestingly, we did not detect interaction between Rab11-DN and Ik2 kinase in the lysates of S2 cells with Rab11-DN and Ik2 co-expression (Fig 5D). However, we indeed detected the formation of Ik2/Spn-F/Rab11-DN ternary complexes in the cells with Spn-F co-expression, but not in the control cells with GFP co-expression (Fig 5D). It implies that Spn-F plays a central role to bring Ik2 and Rab11 together to form Ik2/Spn-F/Rab11 ternary complexes in cells. Without Spn-F proteins, there is no interaction between Ik2 kinase and Rab11 proteins.

Next, we set out to map the Rab11-interacting domains of Spn-F, and determined the role of Spn-F/Rab11 interaction in neurons for dendrite pruning. To identify the Rab11-interacting domains of Spn-F, we used several Spn-F deletion mutants (Fig 5E) [24] and examined their interactions with Rab11 in S2 cells. We found that both SpnF-ΔCC3 and -ΔC proteins retained normal interactions with Rab11-DN (Fig 5E), indicating that removal of the Ik2-interacting CC3 domain [24] and the C-terminus of Spn-F proteins did not affect the Spn-F/Rab11 interaction (Fig 5E). However, the deletion of Spn-F SCD domain completely abolished the interaction between Rab11-DN and Spn-F (Fig 5E), indicating that the SCD domain of Spn-F is critical for Spn-F to interact with Rab11 in cells. Finally, to study the role of Spn-F/Rab11 interaction in C4da neurons for dendrite pruning, we first used transgenic flies to overexpress SpnF-ΔSCD proteins in neurons. In contrast to the control neurons and the ones expressing wild-type Spn-F, the C4da cells expressing SpnF-ΔSCD proteins showed evident dendrite pruning defects at 16 h APF (Fig 5F and 5G), suggesting that SpnF-ΔSCD proteins might function as a dominant negative mutant to interfere the endogenous Spn-F/Rab11 interaction and result in defective dendrite pruning. Next, to validate the role of Spn-F/Rab11 interaction in C4da neurons for dendrite pruning, we performed rescue experiments with SpnF-wt and -ΔSCD in C4da neurons of *spn-F* mutants. Unlike the wild-type Spn-F, SpnF-ΔSCD failed to rescue the dendrite pruning defects of C4da cells in *spn-F* mutants at 16 h APF (S5 Fig) [24], revealing a crucial role of Spn-F/Rab11 interaction in C4da neurons for dendrite pruning. Collectively, our data demonstrate that Ik2, Spn-F and Rab11 can form complexes in cells, and the Spn-F/Rab11 interaction is critical for dendrite pruning in C4da neurons.

### The Ik2/Spn-F complexes promote Rab11 GTPase activation

**Fig 6:**
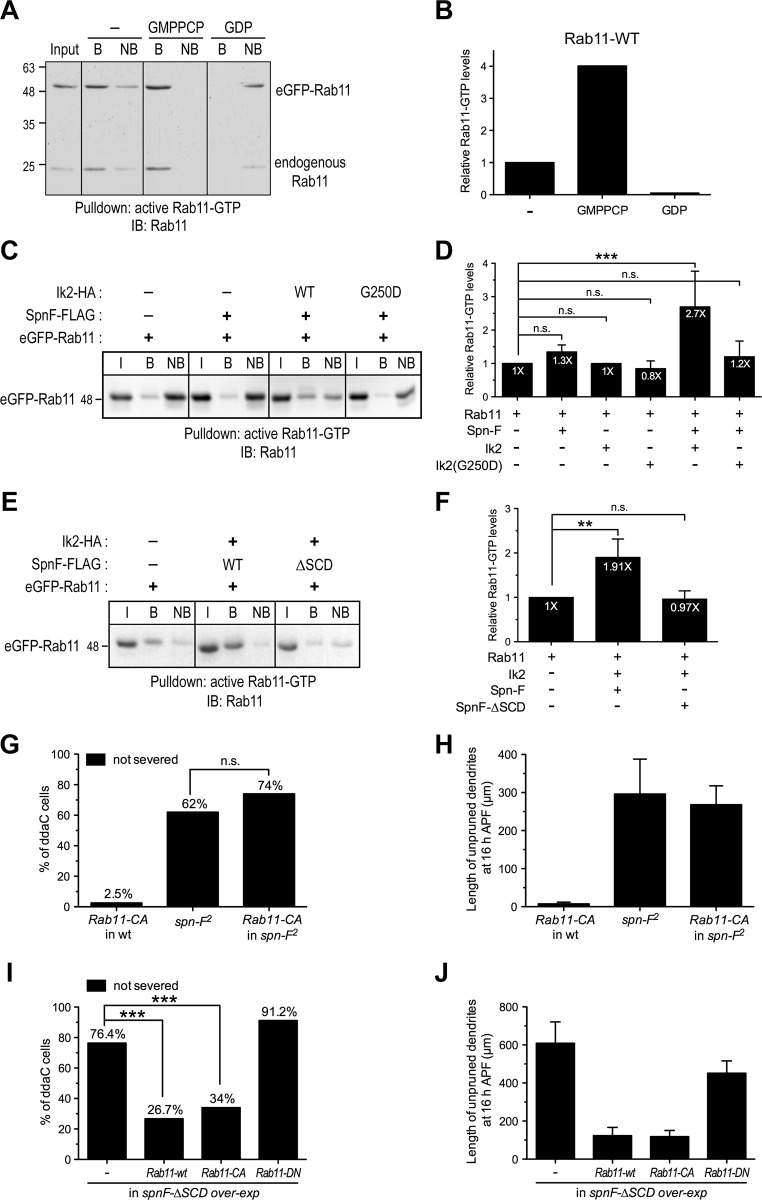
Ik2 and Spn-F act together to promote Rab11 GTPase activation. (A) The same batch of lysates of S2 cells expressing eGFP-Rab11 was divided into three aliquots: no treatment, incubated with GMPPCP (a non-hydrolyzable GTP analog) or with GDP, and subjected to pulldown assays with the antibodies against active Rab11-GTP. (B) Quantification of relative Rab11-GTP levels of eGFP-Rab11 in cell lysates with different treatments shown in (A). The signal intensity of bound eGFP-Rab11-GTP in cell extracts without any treatment was assigned as 1. (C) The active Rab11-GTP antibodies were applied to pulldown eGFP-Rab11-GTP in lysates of S2 cells expressing Spn-F alone, or Spn-F with wild-type Ik2 or with kinase-dead Ik2 (G250D). (D) Quantification of relative Rab11-GTP levels in cells expressing distinct combination of Spn-F, Ik2 and Ik2-G250D. n≥4 for each group. (E) The pulldown assays with active Rab11-GTP antibodies were applied to cell lysates of S2 cells expressing Ik2 and wild-type Spn-F or Spn-F-ΔSCD. (F) Quantitative analysis of relative Rab11-GTP levels in cells expressing various combination of Ik2, Spn-F and Spn-F-ΔSCD. n = 3 for each group. (D,F) One-way ANOVA was performed with a Dunnett’s multiple comparison test (**, p<0.01. ***, p<0.001. n.s., not significant). (G,I) Quantitative analysis of pruning defects in ddaC neurons at 16 h APF. The percentage of ddaC neurons showing pruning defects among the total number of neurons examined. (G) For *Rab11-CA* (constitutively active) overexpression (over-exp), n = 80; for *spn*-F^2^ mutants, n = 60; for *Rab11-CA* overexpression in *spn-F*^*2*^ mutants, n = 90. (I) For *spnF-*Δ*SCD* over-exp, n = 110; for *Rab11-wt* and *spnF-*Δ*SCD* over-exp, n = 120; for *Rab11-CA* and *spnF-*Δ*SCD* over-exp, n = 100; for *Rab11-DN* and *spnF-*Δ*SCD* over-exp, n = 80. Fisher’s exact test was performed in (G), p = 0.1068 (n.s., not significant). Chi-square tests were performed in (I) (***, p<0.0001). (H,J) Quantitative analysis of the total length of unpruned dendrites in ddaC neurons at 16 h APF. (H) For *Rab11-CA* overexpression, n = 40; for *spn-F*^*2*^ mutants, n = 19; for *Rab11-CA* overexpression in *spn-F*^*2*^ mutants, n = 18. (J) For *spnF-*Δ*SCD* over-exp, n = 20; for *Rab11-wt* and *spnF-*Δ*SCD* over-exp, n = 26; for *Rab11-CA* and *spnF-*Δ*SCD* over-exp, n = 32; for *Rab11-DN* and *spnF-*Δ*SCD* over-exp, n = 11. I, input; B, bound; NB, not bound. IB, immunoblotting. Error bars show SD in (D, F); SEM in (H, J).

To further investigate the functional role of Ik2/Spn-F/Rab11 ternary complexes, we asked whether Ik2 kinase and Spn-F could regulate the activity of Rab11. We tried to determine the level of Rab11 activation in cells by performing pulldown assays on cell extracts with an antibody that specifically recognizes the active GTP-bound Rab11 proteins [33]. First, to verify the specificity of this antibody, we employed this antibody to pulldown the active GTP-bound Rab11 from the cell lysates incubated with GMPPCP, a non-hydrolyzable GTP analog, or with GDP. We found that more Rab11 proteins, including both transfected eGFP-Rab11 and endogenous Rab11 proteins, could be pulled down by this antibody from the cell lysates treated with GMPPCP than from the ones treated with GDP and the control ones without any treatment (Fig 6A and 6B). It demonstrates that this antibody has a high specificity to the active GTP-bound Rab11, but not to the inactive GDP-bound one.

Next, to quantify the level of active GTP-bound Rab11 in cells, we applied this Rab11-GTP-specific antibody in pulldown assays on the lysates of S2 cells expressing eGFP-Rab11 together with various combination of plasmids carrying *spn-F*, *ik2* or *ik2-G250D*, which encodes a kinase-dead Ik2 protein. We found that the amount of active GTP-bound Rab11 (eGFP-Rab11-GTP) was significantly increased in the lysates of cells expressing both Ik2 kinase and Spn-F, while compared to that of cells expressing Spn-F alone and of the control ones (Fig 6C and 6D). It suggests that Ik2 kinase and Spn-F together, not Spn-F alone, could promote Rab11 activation in cells. Furthermore, we did not detect increased amount of active Rab11-GTP proteins in the cells expressing Ik2 proteins alone (Fig 6D) or in the cells co-expressing kinase-dead Ik2-G250D and Spn-F proteins (Fig 6C and 6D), implying that Ik2 kinase alone cannot assist Rab11 activation, and the kinase activity of Ik2 is critical for Ik2/Spn-F complexes to promote Rab11 activation. Taken together, these results suggest that Ik2 and Spn-F function together to promote Rab11 activation, and the kinase activity of Ik2 is crucial for Rab11 activation by Ik2 and Spn-F in cells.

Given the SCD domain of Spn-F is critical for the interaction between Spn-F and Rab11 (Fig 5E), we questioned whether this Spn-F/Rab11 interaction is important for Rab11 activation by Ik2 kinase and Spn-F. To answer this question, we quantified the level of active GTP-bound Rab11 in the cells expressing Ik2 kinase with either wild-type Spn-F or mutant SpnF-ΔSCD by pulldown assays. Unlike the cells with Ik2 and wild-type Spn-F co-expression, we did not detect increased level of active Rab11-GTP proteins in the cells with Ik2 and SpnF-ΔSCD co-expression (Fig 6E and 6F). Since the kinase activity of Ik2 is crucial for Rab11 activation promoted by Ik2 and Spn-F (Fig 6C and 6D), and SpnF-ΔSCD still retains the normal interaction with Ik2 kinase [24], these results indicate that the Spn-F/Rab11 interaction is critical for Rab11 activation by Ik2 and Spn-F in cells.

Since our data demonstrate that Ik2 and Spn-F act together to promote Rab11 activation in cells, and the active GTP-bound Rab11 is essential for dendrite pruning, we then asked whether the constitutively active form of Rab11, Rab11-CA, could rescue the pruning defects in neurons of *spn-F* null mutants and in neurons with SpnF-ΔSCD overexpression. After overexpression of Rab11-CA proteins in *spn-F* null mutants, we found that the pruning defects in *spn-F* mutant neurons could not be rescued by Rab11-CA overexpression (Fig 6G and 6H). However, the pruning defects in C4da neurons with SpnF-ΔSCD overexpression could be partially rescued by Rab11-wt and -CA, not by Rab11-DN expression (Fig 6I and 6J). Although SpnF-ΔSCD proteins act as a dominant negative mutant in wild-type C4da neurons, overexpressed Rab11-wt and -CA proteins could still work together with endogenous Spn-F to rescue the pruning defects (Fig 6I). Therefore, the incapability of Rab11-CA to rescue the pruning defects in C4da neurons of *spn-F* null mutants (Fig 6G) could be due to the absence of wild-type Spn-F proteins present in *spn-F* null mutants. These results indicate that increasing the amount of Rab11-CA proteins could partially rescue the pruning defects in the neurons with SpnF-ΔSCD overexpression, but not in the neurons of *spn-F* null mutants. Taken together, our findings suggest that Rab11 activation is likely to be one of the targeting events in Ik2/Spn-F signaling for dendrite pruning of C4da neurons.

## Discussion

Neuronal pruning is a widely observed strategy across the animal kingdom and adapted by the developing nervous systems to refine their circuitry [1]. Pruning is considered as a self-destruction program [2,34], and thus requires tight regulation to confine this pruning activity in restricted cellular compartments. The dendrite pruning of *Drosophila* sensory neurons is a well-characterized model to study the regulatory mechanisms that confine pruning activity in the dendrites. We previously showed that both Ik2 kinase and its downstream mediator Spn-F are essential for the dendrite severing in pupal C4da neurons [11,24]. Moreover, Ik2 is currently the only known molecule sufficient to cause precocious dendrite severing in larval neurons [11], underlining the central role of Ik2 kinase in dendrite pruning. However, how Ik2/Spn-F signaling leads to dendrite pruning in pupal C4da neurons remains unknown. Here we found a small GTPase Rab11 playing an important role and showing a functional connection with Ik2/Spn-F signaling in the dendrite pruning of *Drosophila* sensory neurons.

In this study, we provided evidence to show that Rab11 plays a critical role not only in the dendrite pruning of pupal neurons, but also in the dendrite morphogenesis of larval neurons. The number and length of dendritic branches are significantly reduced in the larval neurons with either *Rab11-dsRNAs* or *Rab11-DN* expression (Fig 2), indicating the crucial role of Rab11 in dendrite morphogenesis. However, the molecular mechanism by which Rab11 regulates dendrite development in fly neurons is unclear. In the case of vertebrate neurons, it is known that Rab11 and its-binding protein Protrudin are both required for neurite formation via directional membrane transport [35]. Although we did not find the homologue of vertebrate Protrudin in fly genome, it is still possible that Rab11 acts together with unidentified proteins, resembling Protrudin in vertebrate neurons, to regulate dendrite morphogenesis in *Drosophila* neurons.

**Fig 7:**
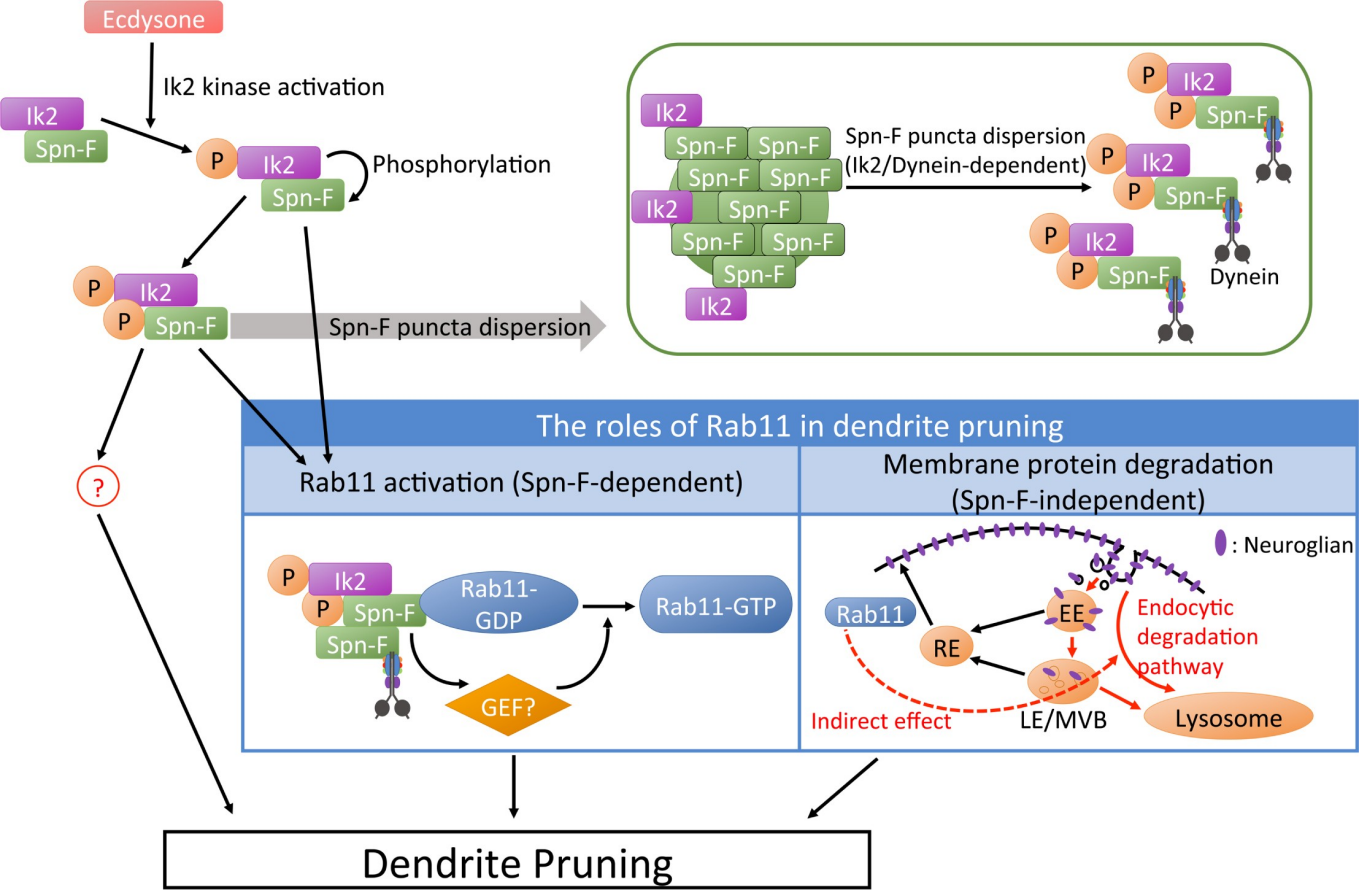
Proposed model for the roles of Rab11 in dendrite pruning. We proposed Rab11 participates in at least two different mechanisms, Spn-F-dependent and -independent, in the dendrite pruning of *Drosophila* C4da neurons. The Spn-F-dependent mechanism is likely involved in Ik2/Spn-F signaling, which consists of Ik2 kinase activation, Spn-F phosphorylation and dynein-dependent Spn-F dispersion. Ik2/Spn-F complexes could promote Rab11 activation through an unknown GEF to promote pruning. Rab11 was previously found essential for Neuroglian degradation in dendrite pruning, and this could be one of the Spn-F-independent mechanisms of Rab11 to mediate pruning. Moreover, we suggested that there could be unidentified downstream targets other than Rab11 in Ik2/Spn-F pathway to regulate pruning. EE, early endosomes; RE, recycling endosomes; LE, late endosomes; MVB, multivesicular bodies.

The results of our GeneSwitch experiments (Fig 2) clearly demonstrated that Rab11 is required for dendrite pruning of pupal neurons. Our studies also showed a modest, but reproducible, genetic interaction between *Rab11* and *spn-F* on dendrite pruning of C4da neurons (Fig 3A). Moreover, we observed more neurons exhibiting pruning defects both in *Rab11*/*spn-F* double mutants and in *Rab11*/*ik2* double mutants than in single mutants (Fig 3B and 3D), confirming the genetic interactions between *Rab11* and *ik2*/*spn-F* on dendrite pruning. Therefore, we proposed two mechanisms, Spn-F-dependent and -independent mechanisms, to elucidate how Rab11 is involved in dendrite pruning (Fig 7).

For the Spn-F-dependent mechanism, considering the genetic interaction between *Rab11* and *spn-F* genes (Fig 3) and normal Ik2 activation in *Rab11*-RNAi neurons (Fig 4), the functional connection between Rab11 and Spn-F might be downstream of Ik2 kinase signaling. It is consistent with our findings in *Drosophila* S2 cells revealing that Ik2 kinase and Spn-F function together to promote Rab11 activation (Fig 6). The activity of Rab GTPases and their cellular functions are regulated by guanine nucleotide exchange factors (GEFs) and GTPase-activating proteins (GAPs) [36]. To promote Rab11 activation, Ik2/Spn-F complexes could either increase Rab11-GEF activity or decrease Rab11-GAP activity. Given the critical role of Spn-F SCD domain in Spn-F/Rab11 interaction (Fig 5E) and in Rab11 activation (Fig 6E and 6F), and the fact that Spn-F has a stronger association with Rab11-GDP than with Rab11-GTP (Fig 5B), we think that increasing the activity of Rab11-GEF is likely the mechanism by which Ik2/Spn-F complexes promote Rab11 activation in cells. Therefore, Spn-F might function as a linker to bring Ik2 kinase, the inactive Rab11-GDP protein and unidentified Rab11 GEF together in a complex. During dendrite pruning in C4da neurons, the Ik2/Spn-F complexes elevate the activity of Rab11-GEF, likely through the kinase activity of Ik2. The activated Rab11-GEF could thereby promote Rab11 activation through exchanging the GDP on the inactive Rab11-GDP with GTP. Once the active GTP-bound Rab11 is formed, it would be discharged from the Ik2/Spn-F complexes and finally lead to dendrite pruning (Fig 7).

The SCD domain of Spn-F protein is critical for Spn-F to interact not only with Rab11-GDP (Fig 5E), but also with Cut up (Ctp) [24], the *Drosophila* homologue of dynein light chain 1 and a subunit of cytoplasmic dynein motor complex. Since both genes *Rab11* and *ctp* are crucial for dendrite pruning of C4da neurons [this paper, 24], the failure of Spn-F-ΔSCD in rescuing the pruning defects of *spn-F* mutant neurons (S5 Fig) [24] is likely a combinatory result caused by disrupting both Spn-F/Ctp and Spn-F/Rab11 interactions concurrently. Given that Spn-F proteins can form oligomers through self-association in cells [24], it is possible that Spn-F interacts with both Rab11 and Ctp concomitantly via the SCD domains of different monomeric Spn-F in the same Spn-F oligomers. It implies that Ik2/Spn-F/Rab11-GDP complexes could link to cytoplasmic dynein via Spn-F/Ctp interaction in the same complexes. If the on-site Rab11 activation by Ik2/Spn-F is required locally in the proximal dendrites, cytoplasmic dynein could be the motor transporting Ik2/Spn-F/Rab11-GDP complexes to the proximal dendrites, where the dendrite severing are expected. However, the cellular site (soma or proximal dendrites) where Ik2/Spn-F complexes promote Rab11 activation in C4da neurons during pruning is currently unclear. Further studies are required in the future.

The degradation of cell adhesion molecule Nrg is impaired in *Rab11* mutant C4da neurons during dendrite pruning [25], suggesting the requirement of Rab11 for normal membrane protein degradation. Since the small GTPase Rab family proteins are the key regulators of intracellular vesicle trafficking and transport networks [37,38], disrupting the function of one member in Rab family might not only directly interrupt specific trafficking pathways, but also indirectly affect other pathways in the networks. It has been reported that the internalization of membrane proteins is reduced in unicellular protozoa *Trypanosoma brucei* upon *Rab11* depletion [39], demonstrating that cells with *Rab11* ablation have a general defect in endocytic pathways. Consistently, the disruption of Rab11 function in C4da neurons could affect general endocytic pathways and indirectly resulted in impaired Nrg degradation during dendrite pruning [25]. Therefore, the defective endocytic pathways caused by impaired Rab11 function could be considered as one of Spn-F-independent mechanisms for the genetic interaction between *Rab11* and *spn-F* genes on dendrite pruning of C4da neurons (Fig 7).

The pruning defects found in C4da neurons with SpnF-ΔSCD overexpression could be partially rescued by Rab11-CA expression (Fig 6I and 6J), suggesting that overexpressed Rab11-CA could still function together with endogenous wild-type Spn-F proteins and lead to dendrite pruning. However, the incapability of Rab11-CA to rescue the pruning defects in *spn-F* mutant C4da neurons (Fig 6G) led us to examine the role of Rab11-CA (Q70L), which has a defective GTPase activity, as a constitutively active Rab11 protein in S2 cells. We performed pulldown assays with the active Rab11-GTP-specific antibody on the lysates of S2 cells expressing eGFP-Rab11-CA (Q70L) (S6 Fig). Unlike the Rab11-CA in *Dictyostelium discoideum* being entirely pulled down by the Rab11-GTP-specific antibody [33], we only pulled down a portion of Rab11-CA proteins in control S2 cell lysates with the same antibody (S6A Fig). Moreover, we could pull down more Rab11-CA proteins in cell lysates incubated with GMPPCP, and less Rab11-CA proteins in cell lysates treated with GDP (S6A Fig). It suggested that the Rab11-CA (Q70L) acts like a wild-type Rab11 in S2 cells (Fig 6A and 6B), and might not function as a constitutively active Rab11 as we expected in C4da neurons of *spn-F* mutants. Consistent with our findings, it has been shown that yeast Rab1 GAP proteins can still accelerate GTP hydrolysis of assumed constitutively active Rab1 proteins, which have the similar glutamine to leucine mutation, both in vitro and in vivo [40–42]. Therefore, it is reasonable to presume that even after GTP binds to Rab11-CA (Q70L) in cells, endogenous Rab11 GAPs could still be able to accelerate GTP hydrolysis of assumed GTPase-deficient Rab11-CA. This could be the possible reason why Rab11-CA (Q70L) proteins could not rescue dendrite pruning defects in *spn-F* mutant neurons (Fig 6G). Another possible reason is that additional unidentified molecules, other than Rab11, in Ik2/Spn-F signaling pathway are also required for dendrite pruning of C4da neurons (Fig 7).

Both *ik2* and *spn-F* have been shown to act in the same pathway during bristle morphogenesis and oogenesis [43,44]. During *Drosophila* bristle elongation, the directional transport of activated Ik2, Spn-F and Rab11 to the bristle tips, where the microtubule minus ends are concentrated, requires Spn-F to act as an adaptor to link Ik2 to dynein complexes [43,45]. Moreover, the activated Ik2 signals and Spn-F are found accumulated at the microtubule minus ends in oocytes and follicle cells during oogenesis [46]. It is similar to our previous findings that both Ik2 activation and dynein complex are essential for Spn-F redistribution, which is crucial for dendrite pruning in C4da neurons [24]. Our new findings that Ik2 kinase activity and Ik2/Spn-F complexes promote Rab11 activation in C4da neurons during dendrite pruning provide a novel molecular mechanism that might be conserved in regulating bristle morphogenesis and oocyte polarity.

## Materials and methods

### Fly strains

The *Rab11*^*ΔFRT*^ [26] was kindly provided by Dr. O. Schuldiner. *spn-F*^*2*^ [30], *ppk-eGFP* [47], *ppk-GAL4* [48] and *UAS-IKKεIR* [31] have been described previously. *UAS-mCD8RFP* was kindly provided by Dr. Y.N. Jan. The following stocks were obtained from Bloomington Stock Center: *UAS-YFP-Rab11* (#9790); *FRT82B*, *tubP-GAL80* (#5135); *UAS-Luciferase-dsRNAs* (#31603); *ppk-CD4-tdTom* (#35845); *GSG2295* (#40266). The *UAS-Rab11-dsRNAs* (#108382) was obtained from the Vienna *Drosophila* RNAi Center (VDRC). The *SOP-FLPs* (#109944, #109945) were obtained from the Kyoto Stock Center. Flies were raised with regular fly foods at 25°C. Transgenic flies *UAS-Rab11-GFP*, *UAS-Rab11-CA-GFP* and *UAS-Rab11-DN-GFP* were generated by standard P-element mediated transformation.

### Evaluation and quantification of the pruning phenotype

Our previous study [11] with ddaC neurons labeled with *ppk-eGFP* or *ppk-GAL4*::*UAS-mCD8GFP* showed that normal dendrite severing occurs around 4–6 h APF, and dendrite debris clearance completes around 16–18 APF. Therefore, we analyzed the pruning phenotype of ddaC neurons at 16 h APF to exclude individual temporal differences. At time of analysis, ddaC neurons having any single dendrite branches with continuous GFP signals extended from the center of soma more than 100 μm were considered pruning defective [24].

### RNAi knockdown efficiency assay

The Rab11-GFP protein was visualized in larval ddaC neurons with *ppk-GAL4*::*UAS-mCD8RFP* and *UAS-Rab11-GFP*. To measure the RNAi knockdown efficiency of *UAS-Rab11-dsRNAs* line, the total fluorescence intensities of GFP in the soma of ddaC neurons were calculated and divided by the soma area to obtain the average fluorescence intensity of Rab11-eGFP of control neurons (expressing *Luciferase-dsRNAs*) and RNAi knockdown neurons (expressing *Rab11-dsRNAs*).

### GeneSwitch experiments

The embryos were collected at intervals of 4 hours and reared on normal fly food until the third larval stages around 96 hours after egg laying (AEL). Then, the larvae were transfer to fly food containing 240 μg/ml mifepristone (Sigma) [21], which was kindly provided by Dr. C.T. Chien, for about 24 hours prior to pupation.

### Molecular cloning

The *spn-F* full-length DNA fragments were amplified from the cDNA clone LD01470 (*Drosophila* Genomics Resource Center). To generate *UAS-spn-F-mCherry* constructs, the *spn-F* full-length DNA fragments were inserted together with mCherry coding sequence into *pUAST* vector. To generate *UAS-spn-F-*Δ*C-myc* constructs, which delete the protein sequences a.a. 340–364 of Spn-F, the deletion DNA constructs were amplified by PCR and inserted together with coding sequences containing myc tag into *pUAST* vector. The full-length DNA fragments of wild-type *Rab11* and two *Rab11* mutants, *Rab11-DN (S25N)* and *Rab11-CA (Q70L)*, were amplified from the plasmids generously provided by Dr. C. Chan. All *Rab11* wild-type and mutant DNA fragments were inserted together with EGFP coding sequence or the coding sequences containing myc, or FLAG tags into *pUAST* vector to make various *UAS-Rab11* constructs. All DNA constructs were verified by DNA sequencing.

### Cell culture, immunoblot and co-immunoprecipitation assays

*Drosophila* Schneider cells (S2 cells) were cultured in Shields and Sang M3 insect medium (Sigma) supplemented with 10% FCS and antibiotics at 25°C. Plasmids were transfected using Effectene (QIAGEN). Cells were transfected with *tubulin-GAL4* to drive various UAS constructs: *UAS-ik2-HA*, *UAS-ik2-G250D-HA*, *UAS-spn-F-myc*, *UAS-spn-F-FLAG*, *UAS-spnF-8A-FLAG*, *UAS-spnF-8D-FLAG*, *UAS-spnF-*Δ*CC3-myc*, *UAS-spnF-*Δ*SCD-myc*, *UAS-spnF-*Δ*C-myc*, *UAS-GFP-myc*, *UAS-myc-Rab11*, *UAS-myc-Rab11-CA*, *UAS-myc-Rab11-DN*, *UAS-FLAG-Rab11-DN*, *UAS-eGFP-Rab11*, *UAS-eGFP-Rab11-DN*. Cells were lysed in lysis buffer (20 mM Tris pH 7.0, 150 mM NaCl, 2 mM EDTA, 1% TritonX-100, 1 mM DTT and protease inhibitors (Roche)). The cell lysates were incubated with anti-FLAG M2 agarose beads (Sigma) on ice and washed thoroughly with lysis buffer. Proteins were eluted and detected using immunoblot assays with rabbit antibody to Myc (Santa Cruz Biotechnology), mouse antibody to FLAG (Sigma), mouse antibody to Rab11 (BD Bioscience, catalog number 610656) and appropriate HRP-conjugated secondary antibodies (Jackson Immuno Research).

### Time-lapse imaging, quantification and image processing

The details of time-lapse imaging of Spn-F distribution in C4da neurons are performed as described previously [24]. In brief, to imaging Spn-F-mCherry distribution in ddaC neurons, larvae and pupae were placed with dorsal side up and imaged with Zeiss LSM 700 laser scanning confocal microscope. To analyze the cytosolic fluorescence of Spn-F-mCherry in neurons, we applied the profile analysis module of the Zen software (Zeiss) to avoid Spn-F-mCherry puncta, which display the peaks of fluorescence intensity. The cumulative fluorescence intensities of dispersed cytosolic Spn-F-mCherry (excluding the signals of any Spn-F-mCherry puncta) were calculated and divided by the pixels numbers to obtain the average dispersed fluorescence intensity in the cytosol of each neuron. The average cytosolic dispersed fluorescence intensity of larval neurons was considered as one to calculate the fold changes of average cytosolic dispersed signals in the same neuron at various time points after pupation. Statistical analysis was conducted and graphs were generated in GraphPad Prism. Images of fluorescent signals were acquired in live animals and fixed samples on confocal microscopes of Zeiss LSM 700. The z-stack images were collected and the maximum intensity projection was used for further analysis. The images were processed using ImageJ (National Institutes of Health), and brightness and contrast were adjusted by Photoshop (Adobe).

### Immunohistochemistry

Primary antibodies used for immunohistochemistry were rabbit anti-P-Ik2 antibodies (1:300, a gift of Dr. S. Hayashi) [32]. The secondary antibodies used for visualizing P-Ik2 signal were Alexa Fluor 488-conjugated donkey anti-rabbit IgG (1:200, Jackson Immuno Research). The larvae and pupae were dissected in cold 1X phosphate-buffered saline (PBS), and then fixed with 4% formaldehyde for 20 min at room temperature. Following fixation, samples were immersed in blocking solution, which is 5% normal donkey serum (Jackson ImmunoResearch). Subsequently, samples were stained with primary antibodies.

### Rab11-GTP activation assay

To measure the activation of Rab11, a mouse monoclonal antibody that specifically recognizes active Rab11-GTP (NewEast Bioscience, catalog number 26919) was employed. *Drosophila* S2 cells expressing eGFP-Rab11 were lysed with lysis buffer. According to manufacturer’s recommended protocol, S2 cell lysates containing equal amounts of total proteins were incubated with anti-active Rab11-GTP antibody for 1 hour at 4°C. The GTP-bound Rab11 was pulled down by protein G magnetic bead (Merck), washed with lysis buffer and eluted by 2X SDS sample buffer. All bound proteins and a portion of proteins from input and not bound fractions were separated by SDS-PAGE and immunoblotted with mouse antibody to Rab11 (BD Bioscience). The proportion of active Rab11-GTP proteins (bound Rab11) to the total Rab11 proteins (bound and unbound Rab11) was calculated for each group from the relative amount of each protein determined by densitometric analysis using ImageJ.

## Supporting information

S1 Fig The localization of Spn-F and organellar markers in neurons. (A-E) Spn-F-mCherry and several organellar markers, including Cnn-GFP (A), Mito-GFP (B), Golgi-GFP (C), Grasp65-GFP (D), and KDEL-GFP (E), were co-expressed in larval C4da neurons under the control of *ppk-GAL4*. *Cnn-GFP* encodes GFP-tagged Centrosomin, which marks centrosome. *Mito-GFP* encodes GFP-tagged human cytochrome c oxidase subunit 8A-derived mitochondrial import sequence, which marks mitochondria. *Golgi-GFP* encodes GFP-tagged human beta-1,4-galactosyltransferase 1-derived Golgi localization sequence, which marks trans-Golgi. *Grasp65-GFP* encodes GFP-tagged Grasp65, which marks Golgi complex. *KDEL-GFP* encodes GFP-tagged endoplasmic reticulum (ER) retention sequence, which marks ER. The organellar markers (A’-E’) and Spn-F-mCherry (A”-E”) manifest as puncta with various sizes. Scale bar, 10 μm.
(TIF)

S2 Fig The knockdown of *Rab11* expression specifically in C4da neurons results in dendrite pruning defects. (A, B) At 16 h APF (after puparium formation), the dendrites were pruned in wild-type (wt) neurons (A), but remained attached to the neurons of *Rab11* RNAi (RNA interference) mutants (B). (C) Quantification of dendrite pruning phenotypes in neurons at 16 h APF. The percentage of cells was determined by dividing the number of neurons with defective pruning by the total number of cells examined for each genotype; for wild type (wt), n = 50; for *ppk-GAL4* and *Rab11-dsRNAs*, n = 90; for *Rab11-dsRNAs*, n = 100. (D) Quantification of the total length of unpruned dendrites in neurons at 16 h APF. For wild type (wt), n = 20; for *ppk-GAL4* and *Rab11-dsRNAs*, n = 43; for *Rab11-dsRNAs*, n = 20. (E, F) The signals of Rab11-GFP were decreased in the soma of ddaC neurons with *Rab11-dsRNAs* expression (F), compared to the wild-type cells with control *Luciferase-dsRNAs* expression (E). (E’, F’) The neurons were visualized with *ppk-GAL4* and *UAS-mCD8RFP*. (G) Quantification of Rab11-GFP intensity in the soma of larval ddaC neurons with control (E) and *Rab11*-RNAi (F) using a two-tailed unpaired t test is shown (***, p<0.001.); for control, n = 9; for *Rab11-*RNAi, n = 10. a.f.u., arbitrary fluorescence units. Error bars show SEM in (D); SD in (G). Scale bars, 20 μm in (B); 10 μm in (F’).
(TIF)

S3 Fig The dendritic morphology of Rab11 mutant larval neurons could be rescued by wild-type or constitutively active-Rab11 expression. (A-E) Confocal images of MARCM clones of larval ddaC neurons. The dendritic morphology is normal in the control clones (A), but abnormal in the *Rab11*^*ΔFRT*^ clones (B). The expression of Rab11-wt (wild-type) (C) or of Rab11-CA (constitutively active) (D), but not Rab11-DN (dominant negative) (E), could rescue the abnormal dendritic morphology in *Rab11*^*ΔFRT*^ clones. Scale bar, 20 μm.
(TIF)

S4 Fig The colocalization of Spn-F with dominant negative Rab11 in neurons. (A-E) Spn-F-mCherry was co-expressed with Rab11-DN (dominant negative) (A), Rab11-CA (constitutively active) (C), in larval ddaC neurons for investigation of colocalization between Spn-F and Rab11 puncta. Colocalizing puncta are defined by showing overlapping signal peaks in signal profile data (B), and non-colocalizing Spn-F puncta are defined by lacking overlapping signal peaks (D). A colocalizing Spn-F punctum (B) and a non-colocalizing Spn-F punctum(D) are demonstrated in Rab11-DN-expressing neuron (A, arrow) and Rab11-CA-expressing neuron (C, arrow), respectively. (E) Quantification of the number of colocalized Rab11/Spn-F puncta in each type of neuron. In the group of small Spn-F puncta (diameter < 0.6 μm), there are significantly more Spn-F puncta colocalizing with Rab11-DN than with Rab11-CA or -wt. Two-way ANOVA with Tukey’s multiple comparison test was performed. *, p<0.05. ****, p<0.0001. n.s., not significant. Error bars show SD. a.f.u., arbitrary fluorescence units. Scale bar, 5 μm.
(TIF)

S5 Fig The Rab11-interacting domain of Spn-F is crucial for dendrite pruning in neurons. (A) Quantification of dendrite pruning phenotypes in neurons at 16 h APF (after puparium formation). The percentage of cells was determined by dividing the number of neurons with defective pruning by the total number of cells examined for each genotype; for control, n = 100; for *spn-F*^*2*^, n = 100; for *spn-F*^*2*^ rescued with *spn-F-wt* (wild-type), n = 88; for *spn-F*^*2*^ rescued with *spn-F-ΔSCD*, n = 100. Fisher’s exact test was performed (***, p<0.0001). (B) Quantification of the total length of unpruned dendrites in neurons at 16 h APF. For control, n = 40; for *spn-F*^*2*^, n = 15; for *spn-F*^*2*^ rescued with *spn-F-wt*, n = 29; for *spn-F*^*2*^ rescued with *spn-F-ΔSCD*, n = 19. Error bars show SEM.
(TIF)

S6 Fig The constitutively active Rab11 in S2 cells. (A) The same batch of lysates of S2 cells expressing eGFP-Rab11-CA (constitutively active) was divided into three aliquots: no treatment, incubated with GMPPCP (a non-hydrolyzable GTP analog) or with GDP, and subjected to pulldown assays with the antibodies against active Rab11-GTP. (B) Quantification of relative Rab11 levels of eGFP-Rab11-CA pulled down by antibody in cell lysates with different treatments shown in (A). The signal intensity of bound eGFP-Rab11-CA in cell extracts without any treatment was assigned as 1. B, bound; NB, not bound. IB, immunoblotting.
(TIF)
