# Supplementary figures and images for: Rab11 activation by Ik2 kinase is required for dendrite pruning in Drosophila sensory neurons
Source: PLoS Genet. 2020 Feb 14;16(2):e1008626. doi: 10.1371/journal.pgen.1008626 (PMC7046344; doi:10.1371/journal.pgen.1008626)

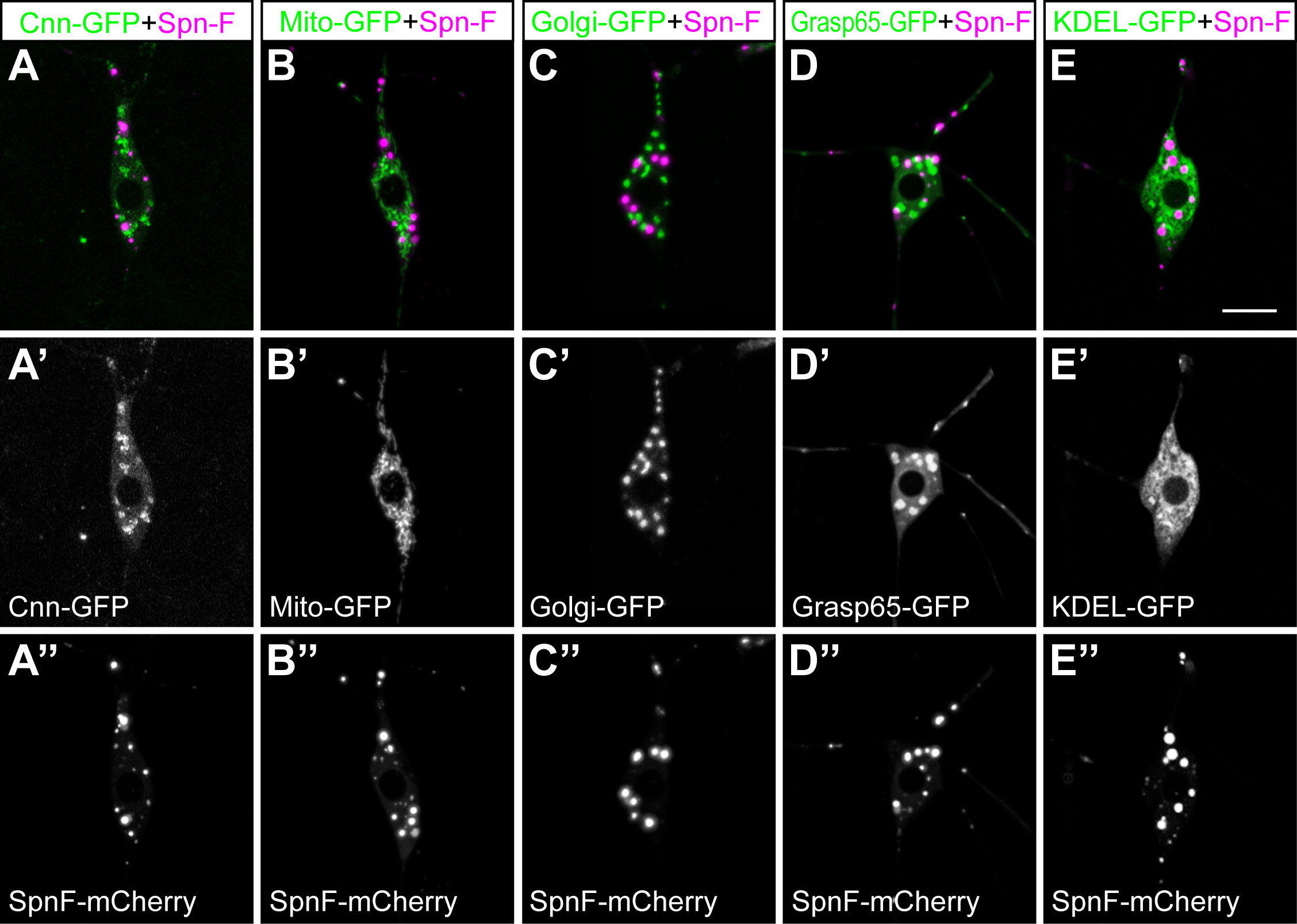

Supplement: S1 Fig — (A-E) Spn-F-mCherry and several organellar markers, including Cnn-GFP (A), Mito-GFP (B), Golgi-GFP (C), Grasp65-GFP (D), and KDEL-GFP (E), were co-expressed in larval C4da neurons under the control of ppk-GAL4. Cnn-GFP encodes GFP-tagged Centrosomin, which marks centrosome. Mito-GFP encodes GFP-tagged human cytochrome c oxidase subunit 8A-derived mitochondrial import sequence, which marks mitochondria. Golgi-GFP encodes GFP-tagged human beta-1,4-galactosyltransferase 1-derived Golgi localization sequence, which marks trans-Golgi. Grasp65-GFP encodes GFP-tagged Grasp65, which marks Golgi complex. KDEL-GFP encodes GFP-tagged endoplasmic reticulum (ER) retention sequence, which marks ER. The organellar markers (A’-E’) and Spn-F-mCherry (A”-E”) manifest as puncta with various sizes. Scale bar, 10 μm. (TIF) [file pgen.1008626.s001.tif]

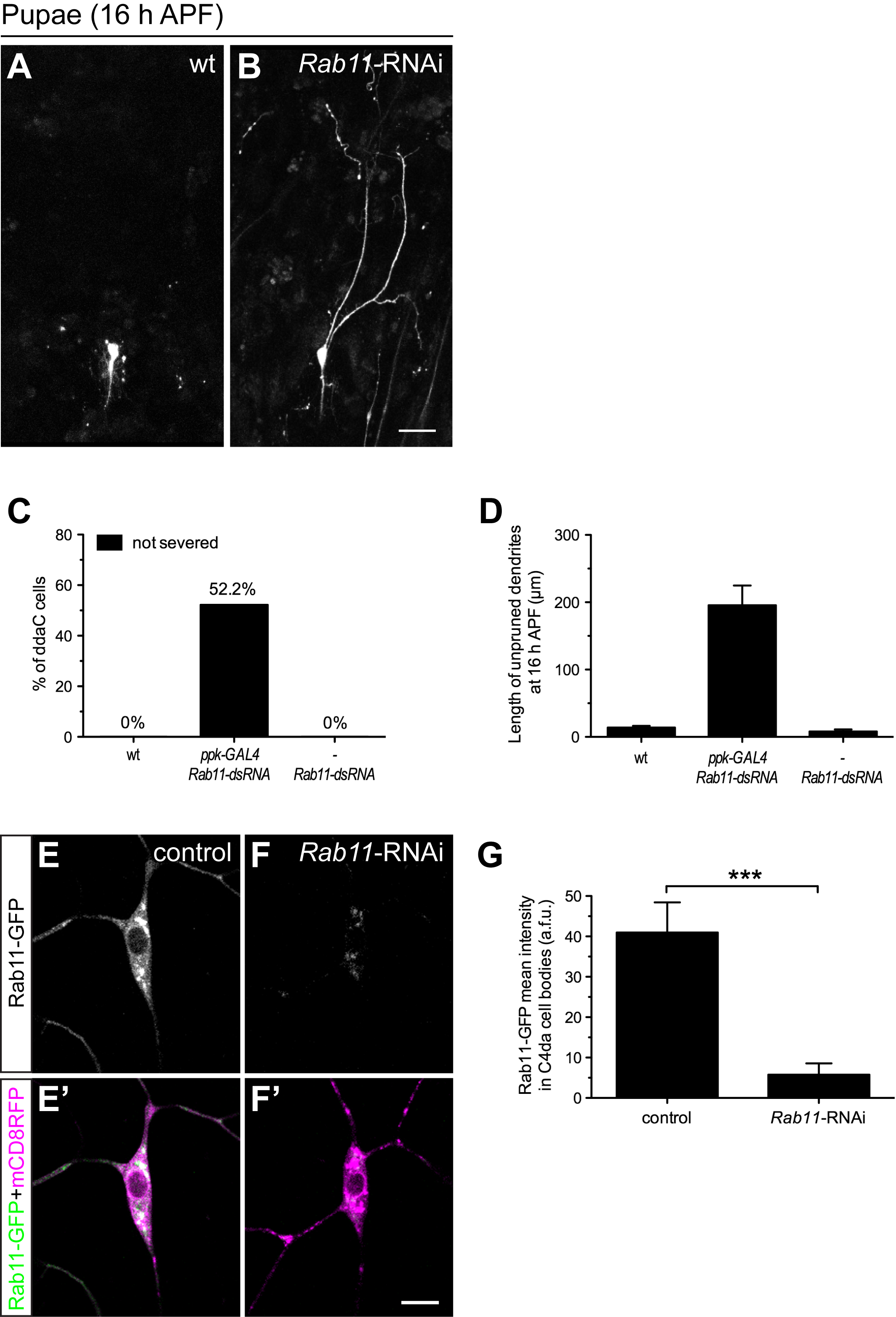

Supplement: S2 Fig — (A, B) At 16 h APF (after puparium formation), the dendrites were pruned in wild-type (wt) neurons (A), but remained attached to the neurons of Rab11 RNAi (RNA interference) mutants (B). (C) Quantification of dendrite pruning phenotypes in neurons at 16 h APF. The percentage of cells was determined by dividing the number of neurons with defective pruning by the total number of cells examined for each genotype; for wild type (wt), n = 50; for ppk-GAL4 and Rab11-dsRNAs, n = 90; for Rab11-dsRNAs, n = 100. (D) Quantification of the total length of unpruned dendrites in neurons at 16 h APF. For wild type (wt), n = 20; for ppk-GAL4 and Rab11-dsRNAs, n = 43; for Rab11-dsRNAs, n = 20. (E, F) The signals of Rab11-GFP were decreased in the soma of ddaC neurons with Rab11-dsRNAs expression (F), compared to the wild-type cells with control Luciferase-dsRNAs expression (E). (E’, F’) The neurons were visualized with ppk-GAL4 and UAS-mCD8RFP. (G) Quantification of Rab11-GFP intensity in the soma of larval ddaC neurons with control (E) and Rab11-RNAi (F) using a two-tailed unpaired t test is shown (***, p<0.001.); for control, n = 9; for Rab11-RNAi, n = 10. a.f.u., arbitrary fluorescence units. Error bars show SEM in (D); SD in (G). Scale bars, 20 μm in (B); 10 μm in (F’). (TIF) [file pgen.1008626.s002.tif]

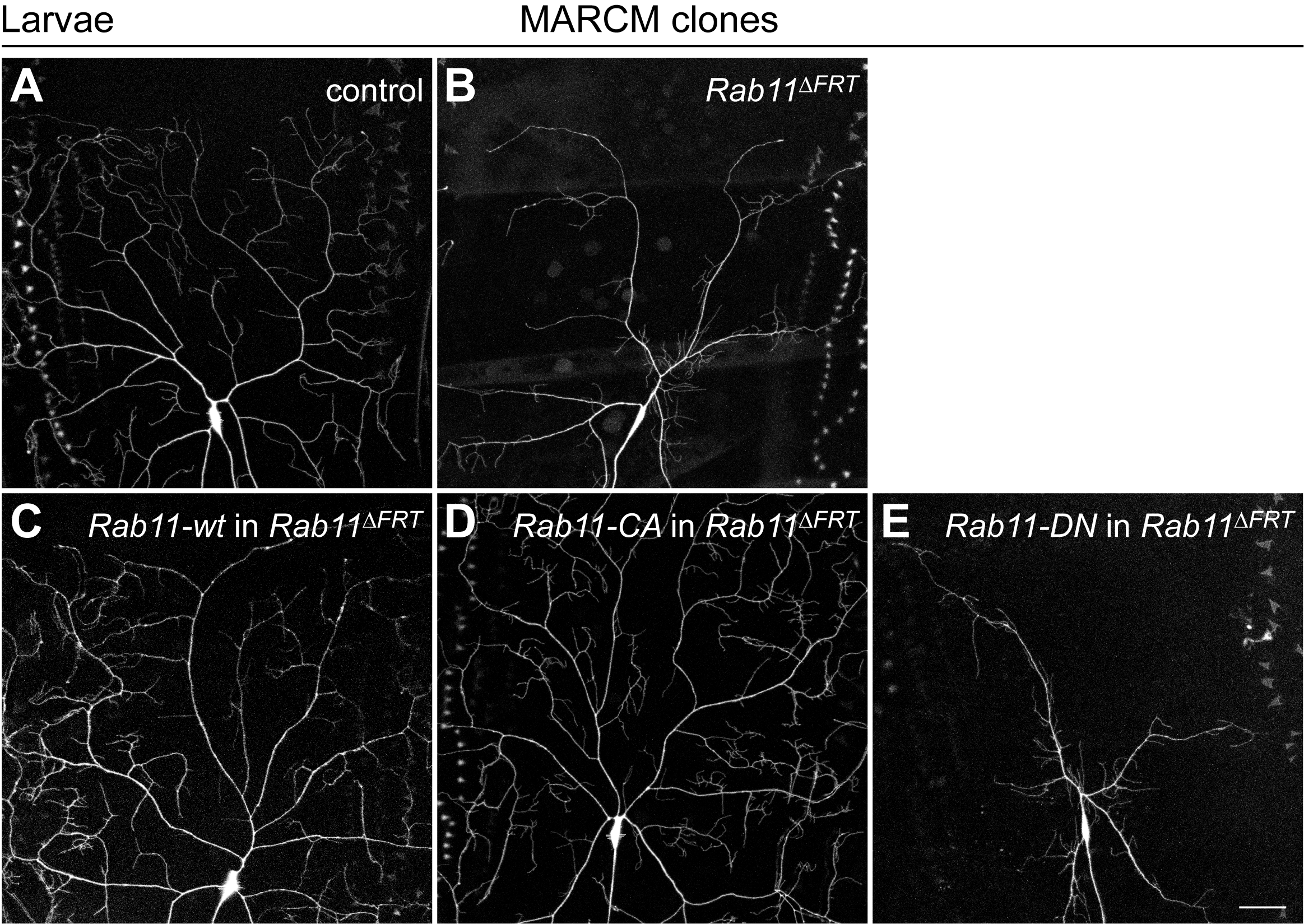

Supplement: S3 Fig — (A-E) Confocal images of MARCM clones of larval ddaC neurons. The dendritic morphology is normal in the control clones (A), but abnormal in the Rab11ΔFRT clones (B). The expression of Rab11-wt (wild-type) (C) or of Rab11-CA (constitutively active) (D), but not Rab11-DN (dominant negative) (E), could rescue the abnormal dendritic morphology in Rab11ΔFRT clones. Scale bar, 20 μm. (TIF) [file pgen.1008626.s003.tif]

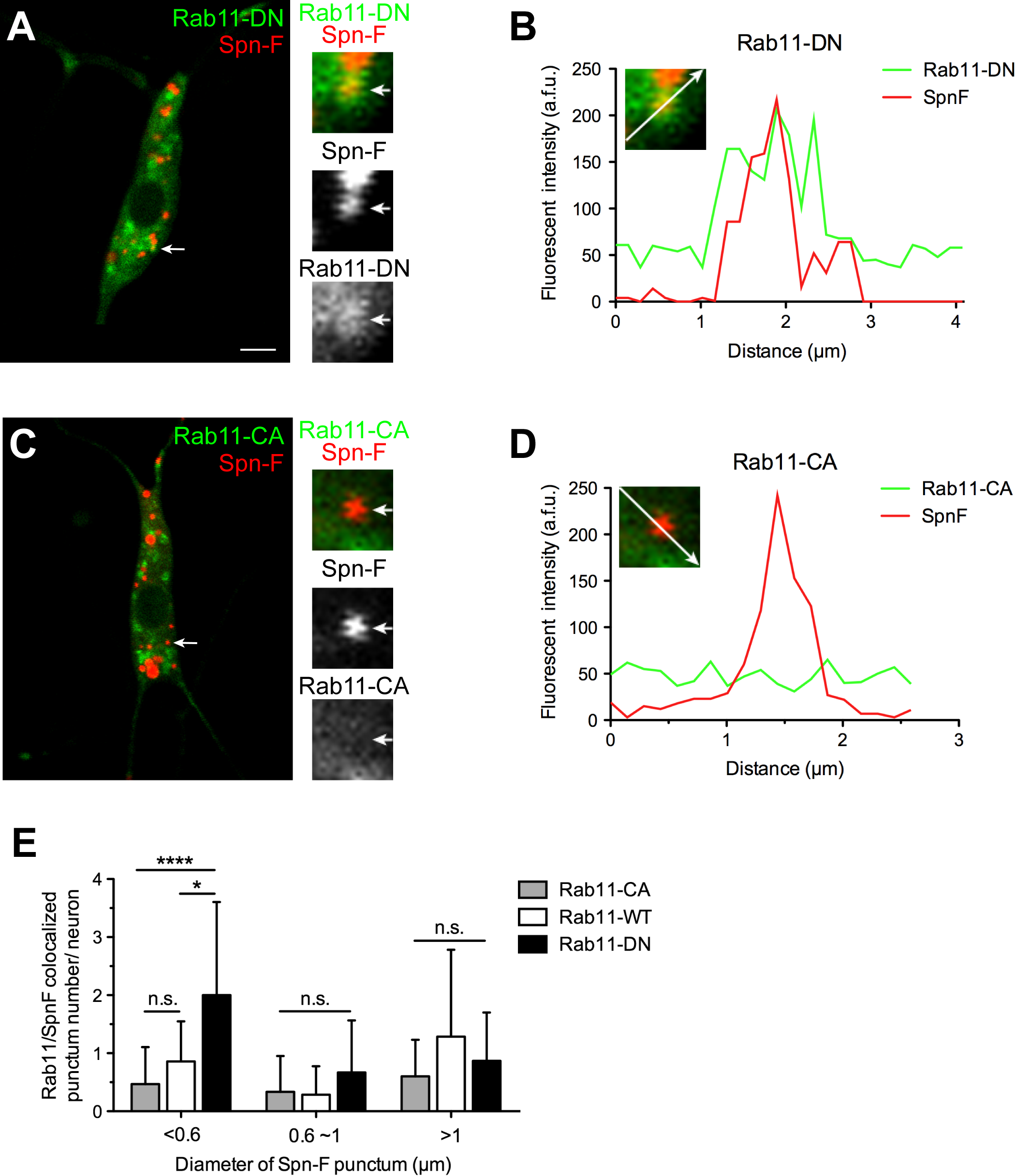

Supplement: S4 Fig — (A-E) Spn-F-mCherry was co-expressed with Rab11-DN (dominant negative) (A), Rab11-CA (constitutively active) (C), in larval ddaC neurons for investigation of colocalization between Spn-F and Rab11 puncta. Colocalizing puncta are defined by showing overlapping signal peaks in signal profile data (B), and non-colocalizing Spn-F puncta are defined by lacking overlapping signal peaks (D). A colocalizing Spn-F punctum (B) and a non-colocalizing Spn-F punctum(D) are demonstrated in Rab11-DN-expressing neuron (A, arrow) and Rab11-CA-expressing neuron (C, arrow), respectively. (E) Quantification of the number of colocalized Rab11/Spn-F puncta in each type of neuron. In the group of small Spn-F puncta (diameter < 0.6 μm), there are significantly more Spn-F puncta colocalizing with Rab11-DN than with Rab11-CA or -wt. Two-way ANOVA with Tukey’s multiple comparison test was performed. *, p<0.05. ****, p<0.0001. n.s., not significant. Error bars show SD. a.f.u., arbitrary fluorescence units. Scale bar, 5 μm. (TIF) [file pgen.1008626.s004.tif]

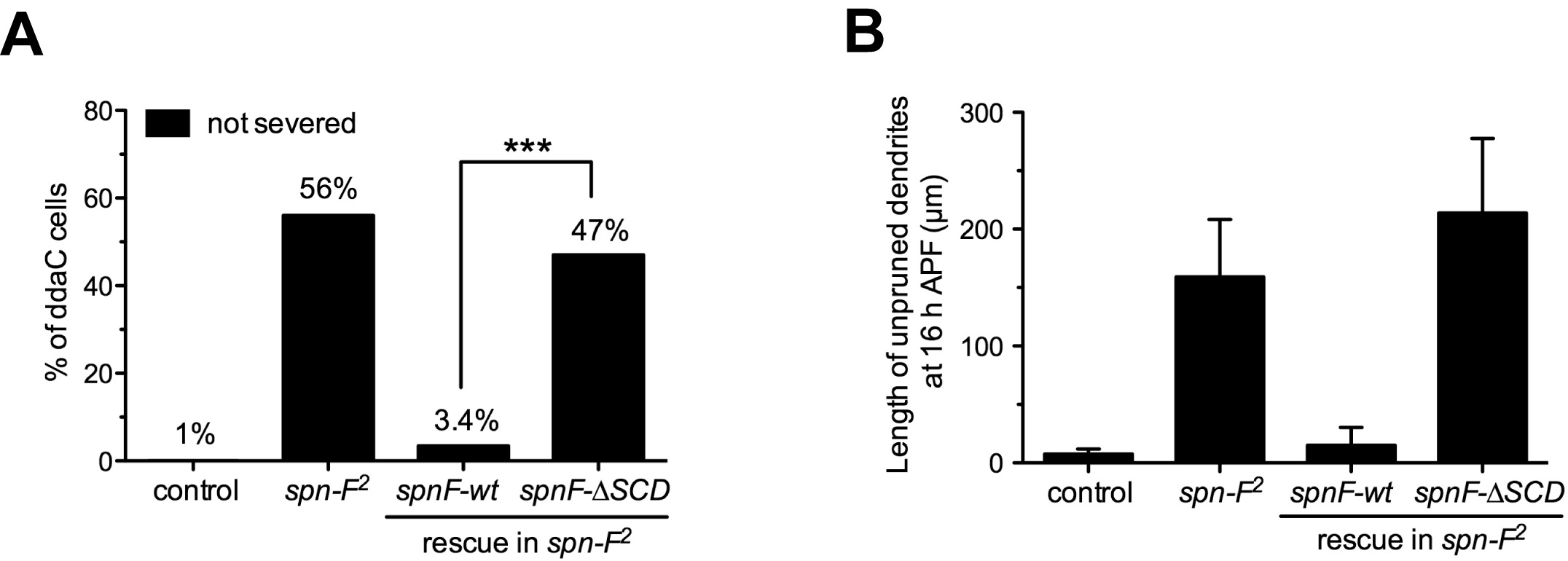

Supplement: S5 Fig — (A) Quantification of dendrite pruning phenotypes in neurons at 16 h APF (after puparium formation). The percentage of cells was determined by dividing the number of neurons with defective pruning by the total number of cells examined for each genotype; for control, n = 100; for spn-F2, n = 100; for spn-F2 rescued with spn-F-wt (wild-type), n = 88; for spn-F2 rescued with spn-F-ΔSCD, n = 100. Fisher’s exact test was performed (***, p<0.0001). (B) Quantification of the total length of unpruned dendrites in neurons at 16 h APF. For control, n = 40; for spn-F2, n = 15; for spn-F2 rescued with spn-F-wt, n = 29; for spn-F2 rescued with spn-F-ΔSCD, n = 19. Error bars show SEM. (TIF) [file pgen.1008626.s005.tif]

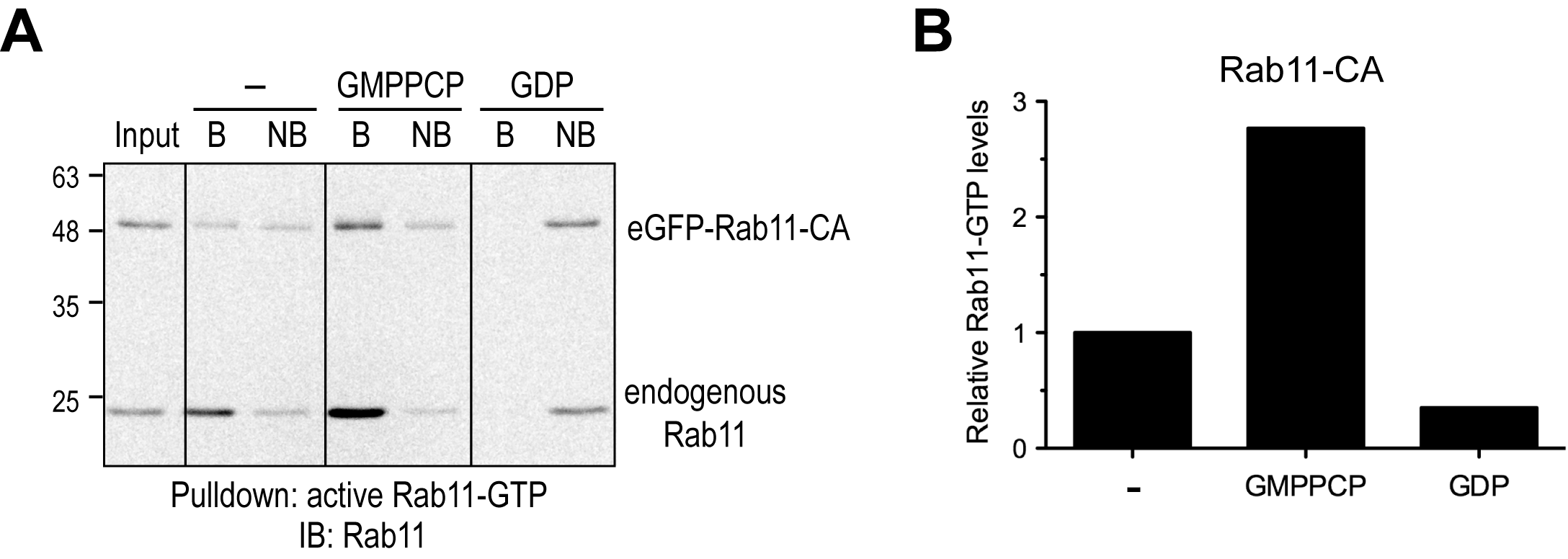

Supplement: S6 Fig — (A) The same batch of lysates of S2 cells expressing eGFP-Rab11-CA (constitutively active) was divided into three aliquots: no treatment, incubated with GMPPCP (a non-hydrolyzable GTP analog) or with GDP, and subjected to pulldown assays with the antibodies against active Rab11-GTP. (B) Quantification of relative Rab11 levels of eGFP-Rab11-CA pulled down by antibody in cell lysates with different treatments shown in (A). The signal intensity of bound eGFP-Rab11-CA in cell extracts without any treatment was assigned as 1. B, bound; NB, not bound. IB, immunoblotting. (TIF) [file pgen.1008626.s006.tif]
